# The Role of the Human Cerebellum for Learning from and Processing of External Feedback in Non-Motor Learning: A Systematic Review

**DOI:** 10.1007/s12311-024-01669-y

**Published:** 2024-02-20

**Authors:** Adam M. Berlijn, Dana M. Huvermann, Sandra Schneider, Christian Bellebaum, Dagmar Timmann, Martina Minnerop, Jutta Peterburs

**Affiliations:** 1 Faculty of Mathematics and Natural Sciences, Heinrich Heine University Düsseldorf, Düsseldorf, Germany; 2 Institute of Clinical Neuroscience and Medical Psychology, Medical Faculty & University Hospital Düsseldorf, Heinrich Heine University Düsseldorf, Düsseldorf, Germany; 3 Department of Neurology, Center for Movement Disorders and Neuromodulation, Medical Faculty & Heinrich Heine University Düsseldorf, Düsseldorf, Germany; 4 Institute of Neuroscience and Medicine (INM-1), Research Centre Jülich, Jülich, Germany; 5 Department of Neurology and Center for Translational and Behavioral Neurosciences (C-TNBS), Essen University Hospital, University of Duisburg-Essen, Essen, Germany; 6 Institute of Systems Medicine and Department of Human Medicine, MSH Medical School Hamburg, Hamburg, Germany

**Keywords:** Cerebellum, Performance monitoring, Reinforcement learning, Cognition, Feedback-based learning, Cerebellar ataxia

## Abstract

This review aimed to systematically identify and comprehensively review the role of the cerebellum in performance monitoring, focusing on learning from and on processing of external feedback in non-motor learning. While 1078 articles were screened for eligibility, ultimately 36 studies were included in which external feedback was delivered in cognitive tasks and which referenced the cerebellum. These included studies in patient populations with cerebellar damage and studies in healthy subjects applying neuroimaging. Learning performance in patients with different cerebellar diseases was heterogeneous, with only about half of all patients showing alterations. One patient study using EEG demonstrated that damage to the cerebellum was associated with altered neural processing of external feedback. Studies assessing brain activity with task-based fMRI or PET and one resting-state functional imaging study that investigated connectivity changes following feedback-based learning in healthy participants revealed involvement particularly of lateral and posterior cerebellar regions in processing of and learning from external feedback. Cerebellar involvement was found at different stages, e.g., during feedback anticipation and following the onset of the feedback stimuli, substantiating the cerebellum’s relevance for different aspects of performance monitoring such as feedback prediction. Future research will need to further elucidate precisely *how*, *where,* and *when* the cerebellum modulates the prediction and processing of external feedback information, which cerebellar subregions are particularly relevant, and to what extent cerebellar diseases alter these processes.

**Supplementary Information:**

The online version contains supplementary material available at 10.1007/s12311-024-01669-y.

## Introduction

Cerebellar contributions to non-motor functions have been extensively investigated over the past decades [e.g., 1–4]. These contributions were highlighted by several consensus reviews and meta-analyses centered on the role of the cerebellum for perception [5], language [6], emotion [7], social cognition [8, 9], and higher cognitive function in general [10]. Cerebellar damage does not only impair (sensori-)motor functions [11, 12], but also affects the cognitive, emotional, and behavioral domains, albeit dependent on the localization and severity of the cerebellar disease [13, 14]. For example, damage to the posterior lobe and vermis of the cerebellum is associated with deficits in executive functions such as task-switching, which was described in terms of the cerebellar cognitive affective syndrome [CCAS: 15, for a meta-analysis see 16].

Neuroanatomical studies revealed multiple neuronal pathways [cerebral-ponto-cerebellar and cerebello-thalamo-cerebral pathways, respectively; 17, 18] as the foundation for functional interactions between the cerebellum and non-motor cerebral areas [19–21]. The functional relationship between cerebellar and cerebral structures was initially conceptualized as a forward model of motor control that was later extended to also apply to the non-motor, cognitive domain [22]. In the motor domain, the cerebellum is thought to underlie sensorimotor integration. According to the forward model, the cerebellum predicts the sensory outcomes of movements based on efference copies of motor commands and adapts behavior based on mismatches between these predictions and the actual sensory outcomes [22–25]. A comparison between intended and actual action consequences is also thought to underlie the processing of performance errors, i.e., when instead of an intended response (e.g., button press with the left index finger) an alternative action is performed (e.g., button press with the right index finger). Cerebellar involvement specifically in error processing has been addressed in some patient studies. These studies provided initial evidence for altered error processing in patients with cerebellar degenerative disease [26, 27 for somewhat conflicting results], and with focal vascular lesions of the cerebellum [28]. Higher cognitive functions such as task-switching and adaptive control of behavior heavily rely on the detection and processing of errors. The cerebellar contribution to error processing may thus be one mechanism by which the cerebellum supports non-motor, cognitive functions.

In many situations, for example in unfamiliar conditions when an individual does not yet know which actions are correct and incorrect, performance errors cannot be identified directly at response onset, or merely based on internal information such as efference copies. In such cases, the individual must rely on external feedback, which can be provided as simple performance feedback (e.g., “correct” vs. “wrong”) or as (monetary) reward or punishment. Here, external feedback can be considered a cognitive consequence of an action, and learning from such feedback for successful behavioral adaptation depends on feedback prediction. Specifically, if the actual feedback does not match the predicted feedback, the behavior needs to be changed. Given its role in generating predictions (see above), the cerebellum may be involved in generating and processing such feedback predictions errors. Indeed, recent evidence on the cellular level in mammals revealed that different cerebellar cell populations were sensitive to reward predictions and reward prediction violations [29, 30]. In this context, the large inhibitory Purkinje cells play a prominent role because they represent the only output neurons of the cerebellar cortex. Their massive dendrite trees in the molecular layer of the cerebellar cortex receive excitatory sensory input from two distinct fiber systems: glutamatergic climbing fibers originating from the inferior olive and glutamatergic mossy fibers that are connected via granular cells to parallel fibers, forming synapses with the dendrite trees of the Purkinje cells. The inhibitory axons of the Purkinje cells project in turn to the deep cerebellar nuclei, which subsequently send excitatory fibers to a broad variety of extra-cerebellar regions. In particular, the ventral part of the dentate nucleus [19] is likely involved in non-motor processes such as predicting feedback by transmitting information to higher cortical structures like the associative regions of the cerebrum [e.g., via the cerebello-thalamo-cortical pathway: 18]. Recent reviews summarize evidence from mammals during learning from feedback [30, 31] that show coding of rewards and reward predictions in several cerebellar cell populations (e.g., granular cells, Purkinje cells, nuclear neurons). It has recently been proposed that cerebellar projections to the ventral tegmental area (VTA) may play a role in reward-based learning [32]. Specifically, these projections modulate the release of dopamine in the VTA, and dopamine is critically involved in coding reward prediction errors and reward value [e.g., 33]. Moreover, dopamine is also linked to movement vigor [34], possibly providing a link between coding of rewards and translation into behavioral output. Cerebellar reward signals that are transmitted to the dopaminergic midbrain and specifically the basal ganglia, a group of subcortical cerebral nuclei critically involved in reward processing and in performance monitoring in general [35], are well in line with the idea put forward by Peterburs and Desmond [36] that performance monitoring may be a core, domain-independent function of the cerebellum.

**Fig. 1:**
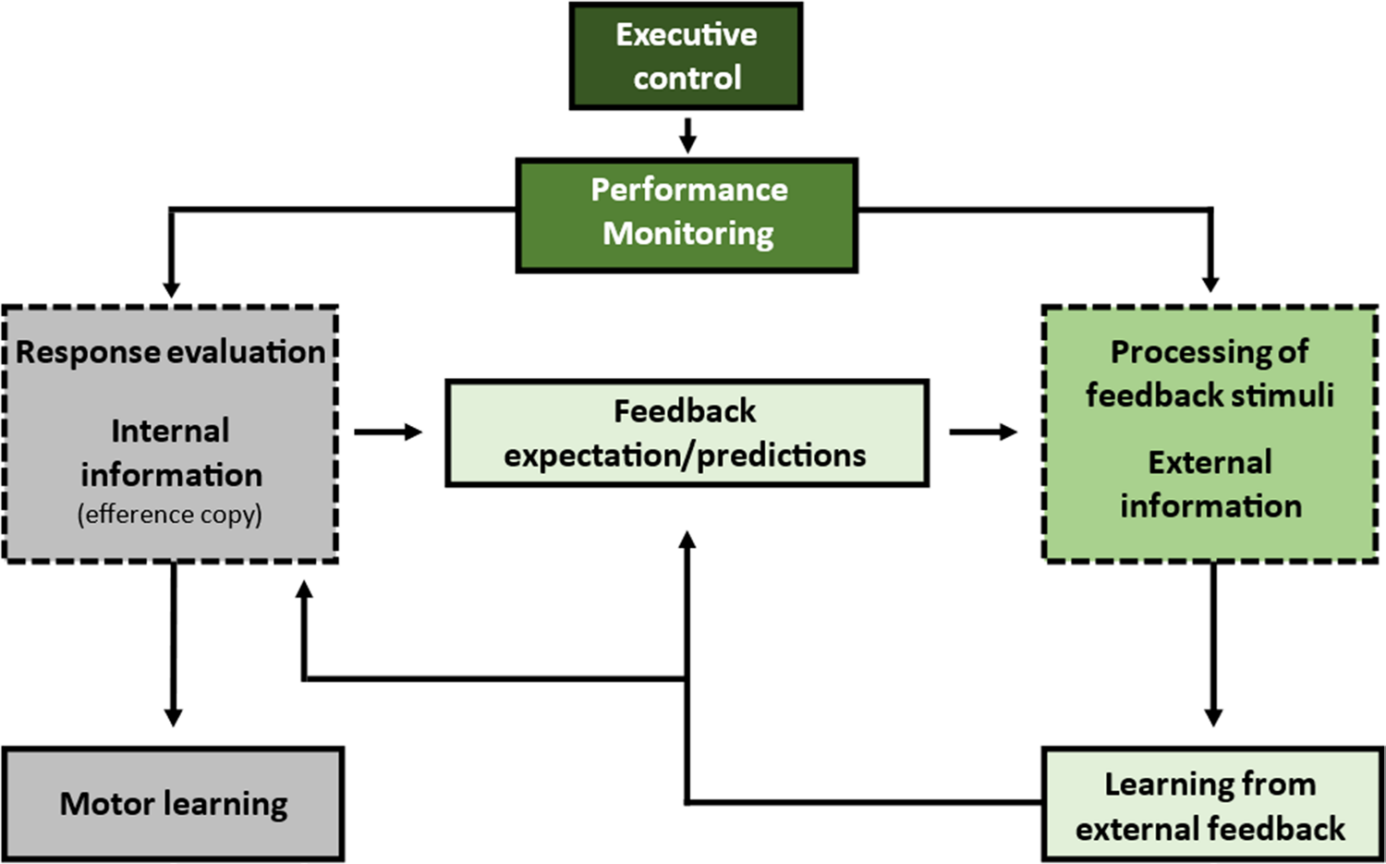
Taxonomy of performance monitoring and key terms and concepts used in the present review. Crucially, the present review is focused on processing of and learning from external feedback in non-motor learning (boxes shaded in greens). Response evaluation based on purely internal information (i.e., efference copies) to optimize motor performance is not addressed (grey boxes)

The terminology used in previous studies on performance monitoring has been inconsistent. For instance, while Frömer et al. [37] use the term “performance monitoring” to describe the internal evaluation of one’s own actions, Peterburs and Desmond [36] define performance monitoring as set of cognitive and affective functions underlying adaptive control of behavior that includes, but is not limited to, error and feedback processing. When reviewing and summarizing the existent work on the cerebellum’s involvement in such processes, it is thus necessary to precisely define these terms. Figure 1 provides a taxonomy and detailed explanation of key terms and concepts in the present review. In this taxonomy, performance monitoring is a subdomain of executive control that incorporates both, the processing of internal information for response evaluation as well as the processing of external feedback stimuli.

**Fig. 2:**
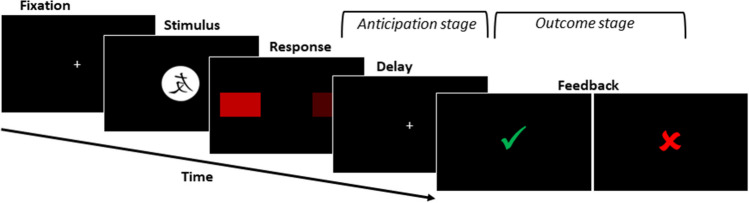
Sequence of stimulus presentation in a generic feedback learning task in which abstract visual stimuli are presented upon which subjects choose between different response options and receive explicit feedback about their choice

The present review focuses on processing of external feedback to enable learning in the non-motor domain. Typical experimental tasks used in this regard include probabilistic learning tasks and reversal learning tasks with abstract visual stimuli [e.g., 38, 39] or a combination of both [e.g., probabilistic reversal learning task; 40]. Figure 2 provides a schematic illustration of the sequence of stimulus presentation in one trial of a generic feedback learning task. After fixation, an abstract visual stimulus is presented, and subjects need to make a response (e.g., press one of two or more response buttons) which is followed by explicit feedback. Over the course of these tasks, feedback predictions/expectations are formed in the period between response execution and feedback presentation (anticipation stage). They are continuously adapted and may directly affect the (neural) processing of feedback stimuli in the outcome stage. Along these lines, brain activation or neural responses that arise in response to cues signaling the impending delivery of specific feedback stimuli reflect feedback predictions [e.g., monetary incentive delay task from 41].

It is also helpful to consider which types of studies can provide insight into the role of the cerebellum in feedback processing and feedback-based learning in humans: First, patient studies, for example in individuals with cerebellar lesions [e.g., 42], can characterize deficits that result from cerebellar damage.

Second, task-based fMRI studies in healthy subjects can shed light on cerebellar activations and cerebello-cerebral interactions associated with the processing of feedback stimuli, e.g., in the context of reversal learning [39]. Regarding the interpretation of cerebellar activations assessed with fMRI, it is useful to keep in mind that granule cell metabolism accounts for most of the energy consumption in the cerebellar cortex [43], and that cerebellar cortical BOLD activation consistently lags behind cerebral activation in connected regions [44]. Thus, the BOLD signal of the cerebellar cortex can be seen as predominantly a reflection of its aggregate input via the ponto-cerebellar pathway. Of note, some previous fMRI studies did not report any cerebellar activations in tasks involving feedback-based learning [45, 46] or excluded the cerebellum entirely from the data acquisition [47]. Also, a review [48] and two activation likelihood estimation meta-analyses covering a broad variety of studies on learning from reward feedback analyzing specific reward types [e.g., monetary, food, erotic: 49] and different stages (e.g., reward anticipation and receipt) and aspects (e.g., valence) of reward processing in adolescents [50] did not report any cerebellar activations.

Third, patient studies can also be combined with other methods, e.g., neuroimaging techniques such as fMRI or electroencephalography (EEG), in order to find out, in how far cerebellar damage affects brain responses to external feedback stimuli. For example, Rustemeier et al. [51] recorded brain activity in cerebellar lesion patients using EEG, assessing specific event-related potential (ERP) components that reflect feedback processing such as the feedback-related negativity [FRN: 52].

Last, studies using non-invasive brain stimulation techniques such as transcranial magnetic stimulation (TMS) or transcranial direct current stimulation (tDCS) applied to the cerebellum in the context of tasks involving processing of and learning from external feedback can also inform about cerebellar involvement in these processes. TMS can be used for both facilitation [53] or disruption of neuronal processes [54]. Most commonly, TMS is used to induce a temporary “virtual lesion” of a target brain area. TMS effects are very localized and can be observed immediately. Single-pulse TMS can be incorporated into fast-paced tasks in a trial-by-trial manner [55]. tDCS effects generally are less localized and build up over time. Since this technique alters the excitability threshold of neurons, it can also be used to facilitate (anodal tDCS) or inhibit activity (cathodal tDCS) in target brain regions [56]. Along these lines, tDCS or TMS applied to the cerebellum can directly manipulate cerebellar involvement in feedback learning and feedback processing. A meta-analysis by Gatti et al. [57] showed moderate effects sizes for cerebellar TMS on responses times and accuracy in different cognitive tasks, e.g., working memory and other tasks assessing executive functions. Regarding tDCS, Mannarelli et al. [58] showed effects of cerebellar cathodal stimulation (compared to sham) on the N2 ERP component in the EEG signal. The N2 is seen as an indicator of response inhibition which was also considered to be a subdomain of performance monitoring [36]. Hence, is stands to reason that this fronto-central ERP component and likely other ERP components originating in cingulate structures in the context of performance monitoring error-related negativity, ERN/Ne: [59, 60] and feedback-related negativity, FRN: [61], can be modulated by non-invasive brain stimulation in a task-based fashion.

In general, the substantial heterogeneity in previous findings, along with the variety of methodological approaches used in the previous works, clearly illustrates the need for a comprehensive review and systematization of cerebellar involvement in processing of and learning from external feedback. To this end, we systematically surveyed and integrated previous findings using a systematic review approach. We included patient studies to address possible alterations in behavioral performance and neuronal activation resulting from cerebellar damage. Studies using neuroimaging techniques such as fMRI and PET were also included if they involved feedback-based learning. Importantly, only studies were included in which external feedback on task performance was presented to enable human subjects (patients or healthy subjects) to adapt and optimize their behavior. Patient studies were restricted to those conducted in individuals with isolated cerebellar disease.

Aside from closing an important gap in the literature, the strength of the present review is the discussion of imaging studies that have not primarily focused on the cerebellum as a region of interest. Indeed, several previous studies collected and reported data on the cerebellum but did not discuss them in detail [e.g., 40, 62], even though these findings may provide important insights into the cerebellum’s role in feedback processing and feedback learning. Ultimately, a more comprehensive understanding of cerebellar contributions to executive functions such as performance monitoring may have direct clinical relevance, as it can help inform, advance, and optimize treatment options for patients with diverse cerebellar diseases.

A preregistration of this review, including a detailed description of inclusion/exclusion criteria and hypotheses, can be found on osf.org (osf.io/2vfg8).

### Hypotheses

In general, we expected findings to support direct cerebellar involvement in processing of and learning from external feedback in a non-motor context. In detail, we expected altered behavioral performance on cognitive feedback-based learning tasks in cerebellar patients. Of note, prior work hinted at the presence of compensatory processes likely relying on structural and/or functional reorganization in patients with chronic, focal cerebellar lesions [28, 63, 64]. We therefore expected altered behavioral performance on cognitive feedback-based learning tasks only in patients with progressive cerebellar degeneration as observed for error processing by Peterburs et al. [26] but not in patients with chronic focal cerebellar lesions.

Based on findings reported by Rustemeier et al. [51], we also expected to find alterations in EEG activity in patients with cerebellar degeneration compared to healthy controls during performance of tasks involving feedback processing and feedback-based learning. Specifically, we expected alterations in ERP components associated with feedback processing [e.g., FRN, 52, 61, 65], P300, [66, 67], and in time–frequency data/oscillations [68, 69]. In addition, fMRI studies conducted in patients with cerebellar damage (particularly due to progressive degeneration) should report altered activation patterns in response to feedback stimuli relative to healthy controls. Unfortunately, we did not find any other electrophysiological studies and no patient study using imaging that fulfilled our inclusion criteria.

Furthermore, we expected fMRI studies in healthy participants to yield activations in the cerebellum and/or in cerebral regions connected with the cerebellum via cerebellar-cerebral networks during tasks involving the processing of external feedback [e.g., 39]. We expected to find cerebellar activations before feedback presentation, thus in the expectation phase, and upon feedback delivery. We also expected feedback-related activity to predominantly involve posterolateral regions of the cerebellum. According to a functional cerebellar topography, these regions are more involved in complex, higher cognitive/non-motor functions [20].

Last, we would expect non-invasive cerebellar stimulation by either cathodal/anodal tDCS [see 70] or TMS (either single or double pulses that are delivered during task performance, or repetitive stimulation prior to task performance) to alter feedback processing and/or feedback learning in healthy subjects.

## Methods

This systematic review followed the guidelines of the PRISMA statement (Preferred Reporting Items for Systematic Reviews and Meta-Analyses [71]. A meta-analysis was not conducted due to diversity of experimental paradigms and heterogeneity in samples and methods. Eligibility criteria were assessed using the PICO framework (Patient, Intervention, Comparison, Outcome framework [72], see Table 1). Beyond the PICO framework, only full-text articles that were primary studies reporting original results (e.g., no reviews/meta-analyses) were included. Moreover, only studies collecting and analyzing quantitative data that were published in peer-reviewed journals and were available in the English language were considered. Studies focusing purely on the sensory and motor capabilities of the cerebellum and studies including patients with extra-cerebellar lesions were not included.

**Table 1:** PICO framework

Population
Humans
Adult participants (≥ 18 years old)
Healthy subjects and patients with a cerebellar disease/lesion
Intervention
Studies using feedback paradigms like: Reversal learning task, Probabilistic feedback learning task,
Wisconsin Card Sorting Test, Weather prediction task, etc
Studies using fMRI, EEG, TMS, tDCS and other methods investigating the cerebellum
Comparators
Healthy and/or clinical comparison groups
Outcomes
Behavioral data: Accuracy, response times
Electrophysiological data: Event-related potentials (ERPs; FRN, ERN, P300), Neural oscillations
Neuroimaging data: Brain activation patterns

Individuals from the healthy control group must not present with any neurologic, psychological, or neuropsychiatric disorder. Individuals from the clinical control group must be diagnosed with a purely cerebellar disease/stroke

### Information Sources

PubMed Database was used to identify relevant articles using the PubMed Advanced Search Builder and the building block approach in which keywords are grouped according to a superordinate term (see Table 2). Further, possibly relevant studies known to the authors were added to the outcome table for the third and final screening round (see below). Only studies that existed prior to the preregistration of this systematic review were used (until 01.07.2021).

**Table 2:** Building block approach for the search strategy

**Concept 1: Cerebellum**
"Cerebellum"[Mesh] OR "Cerebellar Ataxia"[Mesh] OR Cerebellum OR Cerebellar OR Cerebellar ataxia OR Cerebellar hemispheres
**Concept 2: Feedback processing**
"Feedback, Psychological"[Mesh] OR "Formative Feedback"[Mesh] OR feedback OR “Feedback processing*”[tw] OR “reinforcement learning*”[tw] OR “prediction error*”[tw] OR “reward-based learning*”[tw] OR “associative learning*”[tw] OR "reversal learning*" [tw]
**Concept 3: Performance monitoring**
“performance monitoring*”[tw], “action monitoring*”[tw] OR “adaptive behavior*”[tw] OR "rule retrieval*" [tw] OR "executive functions*" [tw]
**Final Search:**
Concept 1 AND (Concept 2 OR Concept 3)

Two additional filters were used within the PubMed environment (Humans, English). The final search took place on the 1st of July 2021, 17:49 CEST with 1057 results

### Search Strategy

To identify relevant search terms, candidate search terms were created and structured according to the building block approach (see Table 2). Search terms were thematically grouped into three distinct key concepts: cerebellum, feedback processing, and performance monitoring. We then manually screened titles in the reference list of Peterburs and Desmond [36] for search terms related to each concept. Each list of candidate search terms was then expanded by adding relevant MeSH (Medical Subject Headings) terms and/or other relevant synonyms related to each key concept (see Table 2). After candidate search terms had been identified, we searched each key concept one at a time by applying an OR operator between search terms, followed by combining all concepts including the respective keywords with the AND operator for the combined search. In addition, search results were filtered in PubMed to only include studies published in English with human subjects. The initial search led to *n* = 839 studies. However, five studies considered relevant and cited by Peterburs and Desmond [36] were not found with this search strategy.

Thus, we added additional keywords to our initial search strategy: Cerebellar hemispheres, reversal learning, rule retrieval, and executive functions. Using the final extended search strategy, we were able to identify *n* = 1057 articles from the PubMed database as eligible for abstract screening. In addition, we now identified three [73–75] out of the five studies which were previously not detected. The building of the final search can be seen in Table 2. Additionally, we added *n* = 21 articles from other sources for abstract screening that were not covered by our search strategy, which led to *N* = 1078 articles that were screened.

### Screening

Abstracts and full-text articles were independently read by two reviewers (A.M.B. and S.S.) using the Abstrackr text‐mining tool [76]. The settings “priority order” and “double selection” were used in the Abstrackr environment. Priority order re-orders the articles starting with the ones with greatest likelihood to be included after each round. Hence, Abstrackr continuously calculated predictions about which articles might be relevant based upon the reviewers’ prior decisions and ordered them accordingly. Moreover, both reviewers used the same screening tool (see supplement Table S1) to first review all available abstracts and then the remaining full-text articles as suggested in the best practice paper by Polanin et al. [77]. The screening tool consists of several questions targeting the most important aspects of the abstract. This was done to ensure that both reviewers kept inclusion and exclusion decisions as objective as possible.

Additionally, we labelled the excluded articles according to the respective number of questions asked within the screening tool. We provided the reason for the exclusion of each article in the PRISMA Flow chart (see Fig. 2). A third (principal investigator J.P.) and fourth (D.M.H.) reviewer were consulted when discrepancies between the assessments of the two initial reviewers were found. The screening process started with a pilot round (*n* = 20 articles) so that questions and problems during the screening could be discussed at an early stage and to ensure that the Abstrackr algorithm was able to sort the articles as intended according to the pilot ratings. Following the pilot round, three main rounds were conducted (first round: *n* = 300, second round: *n* = 300, third round: *n* = 458).

### Extraction

Data collection was combined with full-text screening and performed after all reviewers found a given article eligible. Two independent reviewers (AB and SS) were involved in the data extraction process (extraction tool, see supplement Table S2). To systemize data extraction, we developed a data extraction form (see supplement Table S3). The data extracted by each reviewer were compared, and major discrepancies such as missing details were discussed until resolved. Differences between extracted data in each extraction category emerged in only a few cases (*n* = 6) and were mainly related to the summary of the main and key results. Inaccuracies in the description of the sample size occurred in four cases and were corrected. The extracted raw data for each included study can be found in the supplemental material (Table S9). The collected data were synthesized in a comparative qualitative analysis in accordance with our research goals and hypotheses. Risk of bias was assessed evaluating the described sample size, statistical power (high power allowing the researchers to find and to discuss also small significant effects, if present), and the general methodological quality of the study (e.g., use of appropriate control group or condition, appropriate reporting of descriptive and inferential statistical results, correcting for multiple comparison). A study was additionally assumed to have a lower risk of bias if the hypotheses were preregistered. However, none of the included studies were preregistered. The threshold of statistically significant reported results of each study was *p* < 0.05.

### Risk of Bias

The objectivity of the selection process was ensured by using the questions of the screening tool, which helped the reviewers to systematically approach and assess the abstracts and full-text articles irrespective of their role and status in the research team (PhD student and student assistant) and their prior knowledge of the topic.

### Interrater Reliability

Consensus between raters was continuously assessed throughout the screening and data collection process. Regular meetings were held to ensure that arising questions were addressed during the selection process. In case of discrepancies between raters, these were discussed with the principal investigator until consensus was reached. Interrater reliability was calculated at multiple time points during the screening process using weighted Kappa as well as the percentage of excluded studies. At the end of the selection process, interrater reliability was calculated to assess if the agreement between the raters was sufficiently high. Of note, due to the nature of the prioritizing option in Abstrackr, articles with low likelihood to be relevant were mostly rated in the later rounds of the screening process, which affected the calculation of weighted Kappa [78]. Weighted Kappa calculates the interrater reliability between two or more raters and is therefore affected by the distribution of ratings. We calculated weighted Kappa after each round to see if the screening patterns of both reviewers were consistent and agreement was high (first round = 0.41, second round = 0.50, third round = 0.97). The overall weighted Kappa was moderately high [0.62, 79, see supplement Table S4], while the percentage of agreement between the reviewers was very high (see supplement Table S5).

### Synthesis

For qualitative literature synthesis, selected studies were grouped into patient and non-patient study (including behavioral, electrophysiological, neuroimaging, see supplement Table S6). As we aimed to understand the role of the cerebellum for feedback processing and feedback-based learning across studies with different kinds of samples, studies including only healthy participants were reported separately from studies with patient groups. Importantly, we included and synthesized results from methodologically diverse studies in order to draw the most comprehensive picture possible of the role of the cerebellum for processing of and learning from external feedback in the non-motor domain.

## Results

**Fig. 3:**
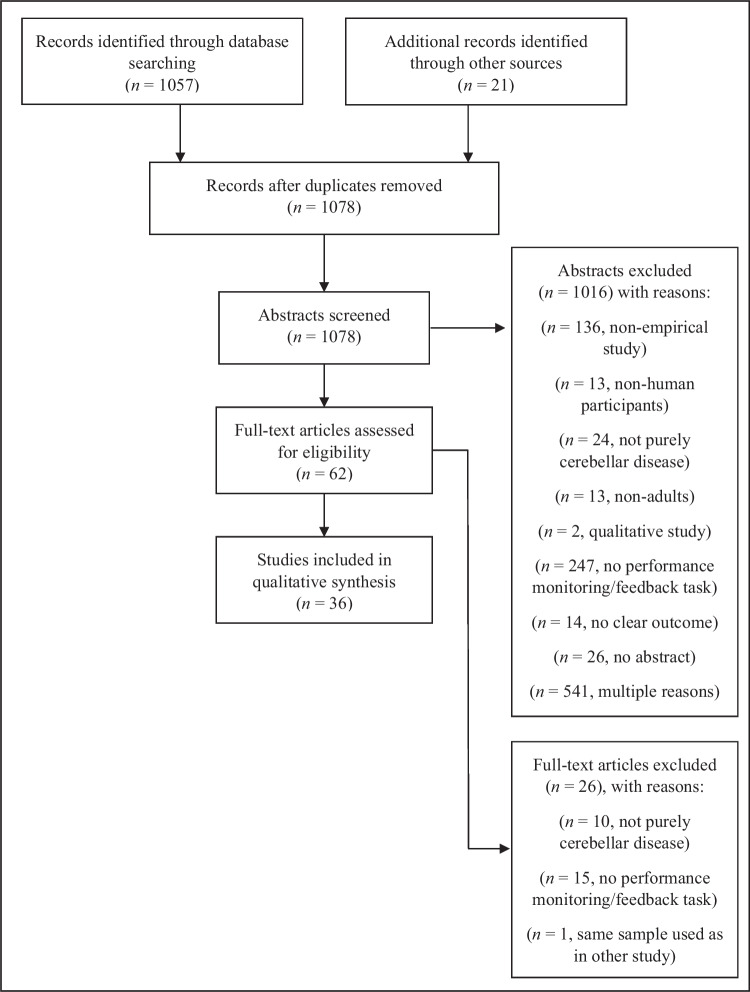
Preferred reporting items for systematic reviews and meta-analyses flow diagram (PRISMA statement)

A total of 1078 abstracts were screened, and 62 articles were selected for full-text reading, leading finally to 36 articles that were included into the review (Fig. 3). The majority of the studies were excluded because they did not include a feedback-based learning task (see Fig. 3 for more details on the exclusion reasons). Among the 36 included studies, we identified *n* = 11 patient studies of which one assessed feedback processing by means of EEG in addition to feedback learning. Of the eleven patient studies, most (7/11) provided data from patients with chronic cerebellar lesions, two included patient samples with cerebellar degeneration [80, 81], one study included patients who underwent neurosurgical resection of tumors located exclusively in the cerebellum [82], and one study included samples with different cerebellar diseases [15], e.g., neurodegeneration, stroke, inflammation of the cerebellum, supplement Table S7). Patient studies included data of a total of *N* = 131 patients. The remaining 25 studies included only healthy participants and were all imaging studies (fMRI:* n* = 22; PET: *n* = 3). A short description of each study can be found in the supplement Table S8.

### Task Descriptions

In the following, a brief overview of the main types of feedback learning tasks used in studies covered by this review is provided. Importantly, all tasks had to contain (trial-by-trial) external feedback that participants could use to optimize their task performance. In the descriptions we will use the terms used by the authors of the studies. These are not necessarily mutually exclusive, as, e.g., a probabilistic learning tasks is also an associative learning task. In our description, we will, however, make clear, what the role of feedback is in these tasks.

In the *Wisconsin Card Sorting Tests (WCST)* and its *modified versions (MCST),* cards depicting geometric objects that differ in properties such as shape, size, and color are drawn from several decks and matched to a sample card. How a card should be matched follows a rule that changes over the course of the test, so participants must monitor and adjust their decisions based on feedback provided by the experimenter that indicates a correct or incorrect choice. Test performance is measured as the number of categories completed, the number of perseveration responses (repeating a certain response option), and the number of perseverative errors (i.e., repeating the error).

In *non-motor associative learning tasks* that include external feedback information [e.g., 80, 81], the association between response options and specific stimuli must be learned by trial and error. A correct choice is indicated by a sound and an erroneous choice by the dis- and reappearance of the stimulus which indicates that a different button must be pressed.

In *probabilistic learning tasks*, a specific case of associative learning, the reward contingencies for a stimulus-response association are not 100 percent so that correct responses are not invariably followed by positive feedback/reward but instead in some cases by negative feedback/non-reward. This increases task difficulty and preserves a degree of exploration behavior. Probabilistic learning can involve *reversal learning* which means that stimulus-response contingencies change throughout the task so that responses that were previously (probabilistically) associated with positive feedback are now associated with negative feedback and vice versa, and response strategies must be adapted accordingly.

In *monetary incentive delay (MID) tasks*, the prediction and anticipation of rewards or punishments can be varied as well as the chance of delivery of the respective outcome (outcome may or may not appear accordingly) indicated by incentive cues reflecting reward probability and magnitude. These incentive cues are presented before the target stimulus and response time window. Performance can be improved based on the provided feedback of winning or losing the indicated reward [for a review on the MID, see 83]. Importantly, studies using this task typically focus on reward anticipation or expectation, e.g., by assessing brain responses to the incentive cues.

### Studies in Patients with Cerebellar Damage

Out of the eleven patient studies, five reported worse performance in the respective cognitive feedback-based learning tasks in cerebellar patients compared to healthy controls [80–82, 84, 85], *n* = 67). Two studies reported no clear evidence for or against cerebellar involvement [15, 86], *n* = 16). Four studies did not find performance differences between patients and controls, or relative to the norm values for the respective versions of the WCST/MCST [87–89], *n* = 36) and no differences between patients and controls in a probabilistic learning task [51], *n* = 12). Importantly, not all patient studies provided the same information on the subscales of the WCST. For instance, the percentage of perseverative errors as an index of deficient feedback-based learning was only reported in five studies (out of eight), and some only provided a mean for the categories completed [84] or z-score of overall performance [15], see Table 3). As outlined above, only five studies reported impaired performance. Deficits were found in feedback learning in patients with cerebellar degeneration [80, 81] who exhibited difficulties in identifying correct associations and needed more time to reach a specific learning criterion as compared to healthy controls. Furthermore, MCST and WCST findings showed fewer completed categories and/or more perseverative errors in patients (MCST in cerebellar stroke patients: [84], WCST in cerebellar lesion patients: [85],WCST in patients with resected cerebellar tumors: [82]. Interestingly, Mak et al. [82] and Mukhopadhyay et al. [85] both reported fewer categories completed and more perseverative errors in patients. However, a significant difference in the percentage of perseverative responses between patients and controls was only found by Mak et al. [82], while the difference was non-significant in the study by Mukhopadhyay et al. [85].

**Table 3:** WCST/MCST results

Study	Year	Task	Categories completed	Peseveration respones	Non-perseverative errors	Perseveration errors	Errors	Overall score
Schmahmann	1998	WCST	"-"	"-"	"-"	"-"	"-"	n.s
Gottwald	2004	MCST	n.s	"-"	"-"	"-"	"-"	"-"
Turner	2007	WCST	"-"	"-"	"-"	n.s	"-"	"-"
Mukhopadhyay	2007	WCST	*P* < C	n.s	"-"	*P* < C	"-"	"-"
Thoma	2008	MCST	n.s	"-"	n.s	"-"	"-"	"-"
Manes	2009	WCST	*P* < C	"-"	"-"	"-"	"-"	"-"
Dirnberger	2010	WCST	"-"	"-"	n.s	n.s	"-"	"-"
Mak	2015	WCST	*P* < C	*P* < C	"-"	*P* < C	*P* < C	"-"

*WCST* Wisconsin Card Sorting Test, *MCST* Modified Card Sorting Test, *P* < C (Controls significantly better than patients), *n.s.* non-significant difference, “- “ = not available

The study by Thoma et al. [86] did not report altered MCST performance in patients with chronic stroke of the cerebellum relative to a matched control group. However, this study revealed a selective impairment in reversal learning based on reward feedback. While patients showed comparable learning success prior to reversal, and better learning of stimuli associated with larger relative to smaller rewards, patients demonstrated poor reversal learning. Moreover, in a subsequent probabilistic learning task, a subsample of patients who were classified as “learners” based upon their prior performance needed more trials to exceed a learning criterion when learning new stimulus-stimulus-outcome associations compared to healthy controls. Rustemeier et al. [51] did not find significant performance differences between patients with post-acute cerebellar lesions and healthy controls in a similar probabilistic learning task.

Despite the lack of performance differences in the probabilistic learning task between patients with chronic cerebellar stroke (*N* = 12) and controls in the study by Rustemeier et al. [51], EEG data revealed significant differences in the ERP. Patients showed higher (i.e., more negative) amplitudes in the FRN for negative compared to positive feedback and a more pronounced (i.e., more positive) P300 for positive compared to negative feedback. In contrast, FRN and P300 were not sensitive to feedback valence in controls. In addition to the initial probabilistic task, the researchers also applied a task with fixed reward contingencies to control for potentially confounding effects of feedback frequency on feedback processing. The results largely replicated the pattern described above. Of note, further analyses appeared to indicate that ERP alterations in patients particularly affected processing of positive feedback, although this effect was not consistently observed in both tasks.

### Neuroimaging Results

**Fig. 4:**
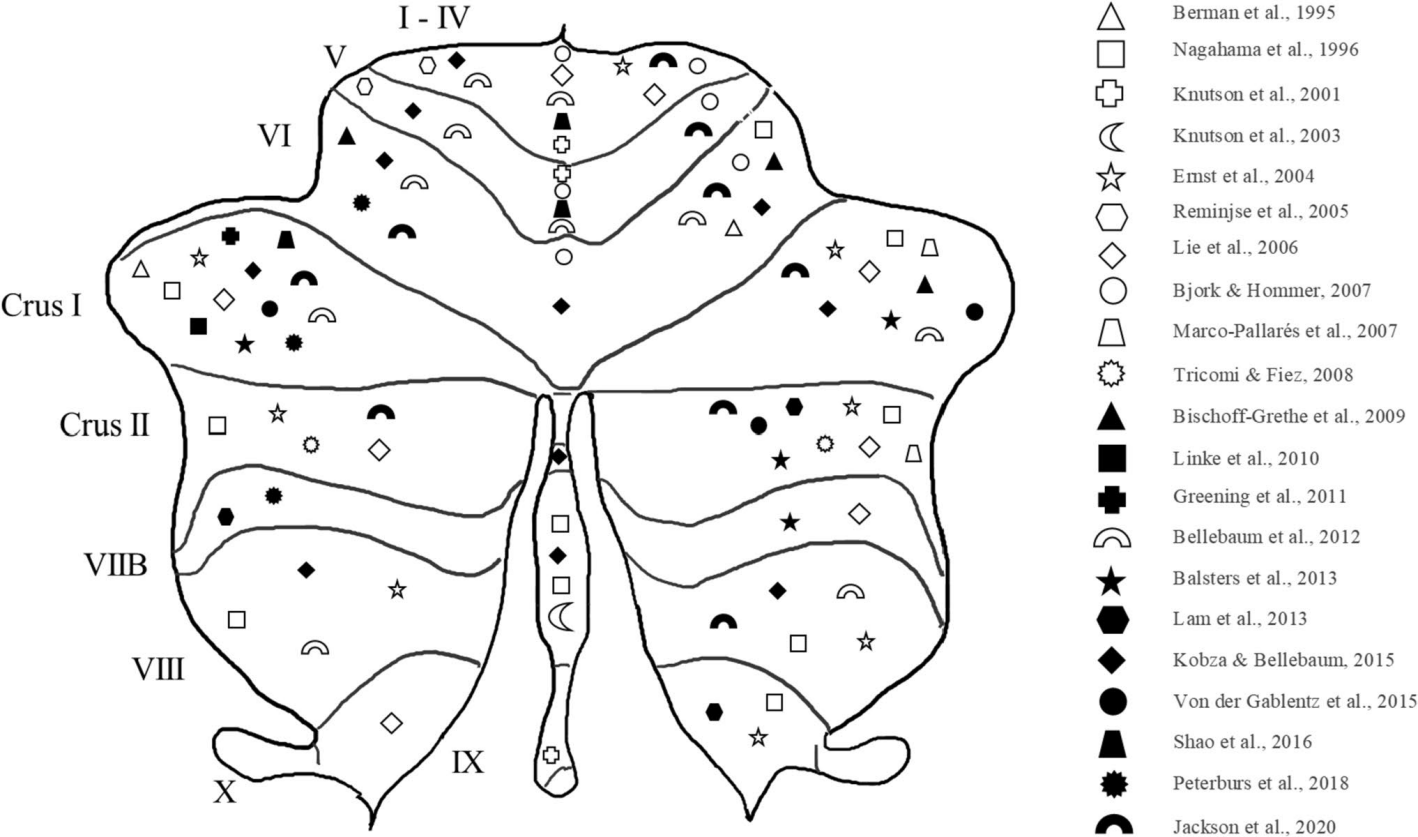
Schematic illustration of the assignment of significant findings from each included imaging study (when coordinates were provided, *N* = 21) to its respective region in a 2D flat map of the cerebellum according to the design in Diedrichsen and Zotow [93]. Importantly, the coordinates in *n* = 4 studies were not provided

All neuroimaging studies were performed in healthy participants (total *N* = 561) but differed with regard to task designs, sample sizes, technical setup, and applied statistical analyses (see supplement Table S8). Importantly, all studies used trial-by-trial feedback for choice behavior. The coordinates of significant cerebellar (peak-) activations for ten studies and the respective analysis were transformed from Talairach into the MNI space using the MNI 2 Talairach Converter program [version 1.2.0, 2020/08/25, 90]. For each study, the coordinates are provided in the supplemental material (see Table S9). To make the distribution of significant cerebellar findings for each study more accessible, we labelled the extracted coordinates using the label4MRI package (version 1.2) in R (R Core Team, version 4.0.3) and RStudio ([91], version 1.3.959) and the AAL atlas taxonomy [92]. Subsequently, each study was assigned a symbol and the significant findings were inserted into a schematic flat map of the cerebellum inspired by the flat map from Diedrichsen and Zotow [93]. Importantly, this figure serves only as a rough illustration and does not represent the exact distribution of the significant voxels in the cerebellum of the calculated contrasts for each respective study (Fig. 4).

The main experimental paradigms in the imaging studies were as follows: seven studies used probabilistic/reversal learning (*n* = 194), three studies used an MID task (*n* = 40), three studies used a non-motor associative learning task (*n* = 35), three studies used WCST/MCST (*n* = 70), two studies used a modified version of a card game (Risk taking task, *n* = 20; Card-guessing task, *n* = 26), one study used a Markov decision task (*n* = 20), one study used a modified version of the dynamically adapted motion prediction task (*n* = 25), and one study used a modified version of the Eriksen-Flanker Task (*n* = 16).

***Substantial bilateral cerebellar activations*** were reported in fourteen studies [38, 42, 73, 75, 94–103], and resting state functional connectivity changes were shown in one study after conducting a task involving rule learning through feedback [104].

Berman et al. [42] contrasted the WCST with a control task (sensorimotor task = matching-to-sample task) and revealed stronger activations in the right (lateral) hemisphere as well as the left posterior hemisphere of the cerebellum. Lie et al. [75] contrasted different executive subdomains in the WCST and discovered increased bilateral cerebellar activations for matching information, error detection, and feedback processing. Ernst et al. [96] found significantly stronger bilateral cerebellar activation (left and right Crus I and lobule VIII, left Crus II) in a risk-taking Task (RTT: gambling card game) compared to a control task involving no decision making. A second run of the RTT also revealed significant bilateral cerebellar activation and right cerebellar peduncle activation when the data on the second run of the RTT were compared to the first run. In addition, Nagahama et al. [101] showed that the bilateral cerebellum was significantly more activated for the MCST relative to a matching-to-sample task. It has to be noted that brain activation in these two studies was averaged across task runs and thus did not reflect exclusively feedback-related activity. In contrast, more recent fMRI work by Tricomi and Fiez [103] did investigate feedback-related brain activations. Increased activation of the bilateral medial inferior cerebellum was found for negative relative to positive feedback trials in feedback-based paired association word-learning task [103]. Interestingly, Bischoff-Grethe et al. [94] reported increased bilateral cerebellar lobule VI activation following positive feedback and right cerebellar lobule VI activation following negative feedback compared to uninformative feedback.

Moreover, Bjork and Hommer [95] showed modulation of cerebellar activations by reward probability in an anticipatory period in which a motor response was necessary: activation in the left vermis IV and V was increased for high vs. low reward probability, and in vermis VI for medium compared to low reward probability. In addition, the right cerebellar lobule VI was active during reward presentation for high compared to low reward probability trials. In accordance with these findings, Lam et al. [100] also found that reward probability of a combination of cards had an influence on cerebellar activations. Here, the right lateral cerebellum was more active for high predictive value vs. low predictive value.

Bellebaum et al. [38] used a probabilistic learning task to contrast active and observational learning. Left lobules IV and V were more strongly activated for expected rewards compared to unexpected rewards in active learners. Observational learners showed increased activation in left lobules IV and V for unexpected feedback compared to active learners. In active learners, only right Crus I was significantly more active for expected non-rewards compared to unexpected rewards, whereas observational learners showed significant activations in bilateral Crus I and II, lobule VI and VIII. Contrasting active with observational learners revealed increased activation in the left lobule IV and V. The reversed contrast yielded more activation in right lobule VI. Activation related to prediction error coding across groups was found in left Crus I and right lobule VI. For active learners only, significant activations were also found in bilateral lobules VIII and right lobule VI. In observers, prediction error related activations were found in bilateral Crus I, right vermis IV and V as well as the left lobule IV and V. These findings thus suggest that the cerebellum may be differentially involved in feedback processing as a function of agency.

This notion was supported by Kobza and Bellebaum [99] who used a different probabilistic learning task (card-guessing task) to contrast active and observational feedback-based learning. Using the uncertainty associated with the card as parametric modulator for fMRI analyses, the researchers found activation in right lobule VI, right vermis VI, and left Crus I in observers. Additionally, when action-independent prediction errors were used as a parametric modulator, significantly increased activation in the right cerebellar lobule VIII and right Crus I were found in the active subsample. Action-dependent prediction errors used as parametric modulators revealed increased activation in right cerebellar vermis VII and left anterior lobules IV/V and left lobules VI and VIII in the active subsample. In addition, the comparison of active against observational for the action-dependent prediction errors demonstrated increased activation in the right vermis VII and left vermis III.

In another card-guessing task [102], participants had to first choose a face-up card out of three and subsequently another face-down card out of three with the instruction to choose the same card as the already determined one. Next, they had to bet credits on whether the cards matched. Feedback was presented as either a win or loss of money. In a second condition, the computer selected the cards and winning or losing was pseudo-randomized, but participants still had to bet credits. Bilateral activation of the cerebellum (labeling according to the provided coordinates: lobules IV and vermis IV and V) was found during the betting stage for the contrast previous winning vs. previous losing outcomes. Shao et al. [102] also reported stronger activation in the left Crus I during the betting stage for computer-generated choices compared to self-generated choices and after previous wins compared to previous losses.

In a modified version of the Eriksen-Flanker task that included reversal learning [97], participants had to respond to a central letter that was surrounded by flanker letters with either a left or right button press. They were informed by feedback if their stimulus-response association was correct or incorrect. The association itself switched across time according to a jittered interval. When a previously correct stimulus-response association switched, the first incorrect feedback was declared as “switch feedback”. Von der Gablentz et al. [97] found increased activation in bilateral cerebellar lobule VIIa for incorrect feedback vs. switch feedback. In addition, the cerebellar vermis was found to be more active for switch feedback relative to correct feedback.

Balsters et al. [73] assessed cerebellar activations during learning of first and second order rules to investigate whether the cerebellum would be engaged only when rules specified the properties of actions (i.e., first order rules = arbitrary stimulus-response mappings), or whether it would also be engaged in learning rules relating to cognitive control independent of action properties (i.e., second order rules which were devoid of motor information). Importantly, this study focused on brain activity in response to instruction cues that specified these rules, rather than the feedback provided in each trial. The most interesting finding in the context of the present review therefore is that the cerebellar lobules Crus I and Crus II were engaged in processing rule-related information irrespective of action properties.

Partially in line with this, Jackson et al. [98] also showed cerebellar activation in a modified second-order rule learning task. Here, the sample consisted of old and young participants. A local peak activation in the right lobule VI was found in older adults for the second order rule in correct trials compared to control trials. Nevertheless, clusters in Crus I and II were also active, but no local peak activation was found. In young adults, bilateral Crus II, right Crus I, and right lobule IV-VI and VIII were activated. Older adults showed more widespread activation compared to young adults. In addition, increased activation of left Crus II and lobules III and VI was discovered in older adults for feedback cues in all learning blocks compared to control blocks. In young adults, areas of peak activation were present in the bilateral lobule VI and bilateral Crus I in response to feedback cues during all learning blocks compared to control blocks.

***Age-related differences*** in cerebellar activity were also demonstrated in a study by Edde et al. [104] in which a modified version of the dual-task paradigm [105] was used. Resting state functional connectivity (rsFC) data were acquired before and after the task with the cerebellum as a region of interest. Young adults (18–30 years of age) demonstrated post-learning activation changes within 44 pairs of brain regions. Forty-two pairs were connections of cerebellar with non-cerebellar regions. Distinct cerebellar networks were fronto-cerebellar, temporo-cerebellar, cerebello-cerebellar. Older adults (61–70 years of age) on the other hand showed fewer learning-related changes in rsFC than young adults and no involvement of cerebellar networks.

***Activations restricted to the vermis*** were discovered in three studies [41, 106, 107]. Späti et al. [107] found increased vermis activation for losses relative to gains in a motion prediction task in which the reward contingencies were fixed. Knutson et al. [41] demonstrated significant activation in the cerebellar vermis for large vs. small rewards/punishments and found significant vermis activation during the anticipation of potential gain vs. no outcome and potential loss vs. no outcome in a subsequent study [106].

***Activations restricted to the left cerebellum*** were found in five studies [39, 40, 62, 108, 109]. For reversal learning in probabilistic feedback tasks, three studies showed increased activation of the left cerebellum for affective switching, i.e., the inhibition of responses towards the previously rewarding stimulus that were now punished and the execution of responses towards the new rewarding stimulus compared to the baseline [40, 62, 108]. Moreover, using a reversal learning task, Peterburs et al. [39] found left-sided activations in lobule VI and VIIa.

Tanaka et al. [109] investigated reward-based learning in terms of predictions and prediction errors using a Markov decision task. In this task, one of three shapes was presented, and participants had to respond with either left or right button press. Feedback was provided as a monetary win or loss. The left lateral cerebellum was activated for future relative to immediate reward predictions. Also, increased activation of the medial cerebellum during immediate reward prediction was found.

***Activations restricted to the right cerebellum*** were found and described in two studies [105, 110]. Marco-Pallarés et al. [110] reported significant cerebellar activations in right Crus I and II for positive compared to negative feedback. Rule information was manipulated in a dual-task study [105] in which a conditional learning task and a verb-generation task were both conducted simultaneously. Significant activation was found in right Crus I for highly informative cues, and a trial-by-trial analysis revealed that this activation decreased faster as learning progressed. In contrast, cerebellar activation in Crus I in response to less informative cues did not decrease with learning progression.

## Discussion

The main goal of this systematic review was to identify and summarize findings pertaining to cerebellar involvement in processing of and learning from external feedback in a non-motor context, following the guidelines of the PRISMA statement [71]. Thirty-six studies met our criteria and were included. Among these were several patient studies, one of which addressed altered electrophysiological activity during feedback processing in patients with cerebellar lesions, and a larger number of fMRI imaging studies (either task-based studies or studies assessing cerebello-cerebral functional connectivity changes associated with feedback learning) conducted in healthy subjects. We did not find any study that used non-invasive brain stimulation techniques to target the cerebellum in the context of feedback-based learning tasks published prior to July 2021, i.e., prior to the preregistration of this systematic review.

### Feedback Learning Performance in Patients with Cerebellar Diseases

The reviewed studies were very heterogeneous regarding tasks and sample characteristics. Likewise, findings were inconsistent: five patient studies reported impaired learning in cerebellar patients [80–82, 84, 85], *n* = 67), while four studies did not find performance differences between patients and controls [51, 87–89] *n* = 48), and two studies reported mixed findings [15, 86], *n* = 28). Aggregating patient samples within these three groups of studies yielded comparable overall sample sizes, further hampering a clear statement regarding the presence or absence of alterations of feedback-based learning in patients. Even within one single study, not all patients demonstrated consistent deficits, as outlined by Tucker et al. [81). Most patients had presented with cerebellar strokes with long intervals between lesion onset and study participation, and this passage of time may have allowed for some functional reorganization. In line with this, Schmahmann and Sherman [15] described improved or normalized executive task performance in “chronic” compared to “acute” cerebellar focal lesions. In addition, it has been shown that targeted rehabilitation may allow for substantial compensation regarding motor [111, 112] and cognitive deficits [113–115]. In contrast, cognitive performance in patients with neurodegenerative cerebellar diseases likely decreases with disease progression, similar to motor symptoms in different types of cerebellar ataxia [116–118]. Aside from time since lesion, lesion location in cerebellar stroke, and severity of cerebellar degeneration, other factors such as the age at lesion onset [119] have also been linked to the severity of cognitive deficits [see 120 and 121 for an overview on stroke related factors].

Only one of the included patient studies recorded electrophysiological data to assess feedback processing [51]. While behavioral data in this particular study did not show differences between cerebellar lesion patients and controls regarding learning from external feedback, the ERP components FRN and P300 indicated altered neural processing of negative and positive feedback in patients. Rustemeier et al. [51] concluded that these altered ERP patterns reflected impaired outcome prediction, although somewhat contrary to this notion, learning performance in patients was similar to controls. Given that in most patients, several years had elapsed between stroke onset and study participation (see supplement Table S7), functional reorganization and/or compensatory processes might help explain this discrepancy.

There is indeed evidence from various studies in cerebellar stroke patients that ERPs, in particular the P300 component, reflect functional improvement over the course of the disease [122–127]. However, the P300 in these cases was not obtained in feedback-based learning tasks. Additionally, stroke patients who recovered best from the injury demonstrated more symmetrical distribution of the EEG power spectrum compared to patients with poorer recovery across a period of six months (period between the stroke onset and the first examination was on average 28.16 days, *SD* = 7.15 days; [128]. Taken together, these findings suggest that EEG in general, and ERPs in particular, might be a useful tool to track changes in neural processing that occur during immediate post-stroke recovery, also in the context of cerebellar lesions.

### Cerebellar Activations in Neuroimaging Studies

We reviewed functional imaging data of 25 studies (total sample of *N* = 561), all in healthy participants, demonstrating activations in the cerebellum and cerebellar-cortical networks during and after tasks involving feedback processing and feedback-based learning. Meta-analyses of functional imaging data with the cerebellum as the region of interest [10, 20], data on functional connectivity [129] or a combination of task-based and functional connectivity data [130], and task-specific parcellation [131] of the cerebellum provide the foundation for interpreting the different results. Buckner et al. [129] demonstrated functional coupling between lobules VI and VII and cerebral networks involved in cognitive control. Stoodley and Schmahmann [20] showed that bilateral Crus I, left lobule VI and VIIB were most active in tasks requiring executive control. Consistent with this, Keren-Happuch et al. [10] reported that the bilateral Crus I, left Crus II, right lobule VI and midline lobule VII were most active during executive processing. More recently, King et al. [131] parcellated the cerebellum into task-specific regions, but clear differentiation of executive tasks with a focus on feedback processing was lacking. The present review attempts to fill this gap, identifying studies with increased cerebellar activation while performing tasks involving processing of and learning from external feedback (e.g., WCST, MCST, RTT) compared to control tasks or conditions that control for several aspects of the respective version of the task [42, 75, 96, 101].

Our review of imaging findings found significant activation in bilateral Crus I and II (see Fig. 3 and Table S9) associated with feedback learning. For instance, Balsters and Ramnani [105] showed significantly increased activation of Crus I for “high learning cues” in which feedback information always reflected the performance in the current trial compared to “low learning cues” which did not. Performance under dual task conditions improved over time, which was interpreted as automatization of rule learning. Likewise, Balsters et al. [73] showed increased activation of Crus I and II during rule learning, highlighting that the posterolateral cerebellum was engaged in processing external, rule-related information irrespective of action properties which is in line with its function as a “prediction machine” within the forward model.

According to our findings, imaging data obtained in non-motor associative learning tasks underlined the significance of several aspects of feedback. First, the context of feedback is important. Tricomi & Fiez [103] investigated whether brain activation patterns differed for feedback that was informative but only arbitrarily related to performance compared to feedback that provided information about goal achievement. Regarding the cerebellum, the most interesting finding was that activations for negative relative to positive feedback were increased when feedback was tied to goal achievement. Second, feedback valence has been shown to differentially activate the cerebellum [e.g., 110]. Interestingly, positive compared to uninformative feedback was associated with increased bilateral activation in cerebellar lobule VI, and right cerebellar lobule VI was significantly more activated for negative compared to uninformative feedback demonstrating the significance of feedback information content [94]. These latter results may be taken to suggest that information content rather than valence is driving cerebellar activity. Along these lines, it could be speculated that the cerebellum may filter out irrelevant information before calculating the respective prediction.

In terms of expectations and prediction errors, previous feedback experiences may affect the anticipation of upcoming feedback, as has been shown for the electrophysiological indices FRN and P300 [132] as well as for the activity of several non-cerebellar brain regions including the anterior cingulate cortex (ACC) and basal ganglia [133, 134]. In this regard, Knutson et al. [41, 106] manipulated the anticipation of reward and punishment size (large vs. small and gain vs. no outcome) and demonstrated that both were associated with increased activation of the cerebellar vermis. This is in line with several studies demonstrating that particularly unexpected feedback was associated with significantly more activation in the cerebellum and suggests that the cerebellum is involved both during feedback prediction and the processing of prediction errors [40, 62, 97, 108].

In addition, cueing the certainty of feedback as a manipulation of expectancy yielded increased vermal activation and stronger right cerebellar activation during the processing of certain wins compared to certain losses. In a somewhat similar manner, higher predictive values of card combinations compared to lower ones led to stronger activation of the right lateral cerebellum [100]. Hence, the anticipation of an outcome could be modulated by cerebellar structures at an early stage before external feedback information is available. Evidence for early processing of feedback information was already found in the stimulus-preceding negativity, a negative slow wave in ERP that occurs before feedback presentation and is suggested to reflect the anticipation of meaningful information [135, 136]. However, no study has yet investigated whether the cerebellum may contribute to the stimulus-preceding negativity, which might be conceivable considering the cerebellar forward model.

Shao et al. [102] showed increased bilateral cerebellar activations (lobules IV) in the betting stage of a card-guessing game when subjects had won in preceding trials compared to when subjects had lost in preceding trials. Moreover, stronger activation in left Crus I was present when outcome expectation had to be articulated into a distinct value, and when participants had experienced more previous self-executed choices and previous wins. Interestingly, the effect of the interaction of agency (either the participant or the computer made the card selection) and outcome (positive or negative feedback) was stronger for computer-generated choices than self-generated ones, particularly after winning compared to losing, suggesting that the cerebellum is also involved in processing agency as a factor determining the optimal decision. Somewhat in line with this notion, action dependent and independent outcome prediction errors were associated with increased cerebellar activity in a subsample of active learners and for action dependent prediction errors when compared to observers [99]. In addition, predictions of future compared to immediate rewards were again associated with activation in the left lateral cerebellum, revealing that the time scale of the reward had an influence on how the cerebellum generated the prediction [109]. Also, feedback valence is an important aspect for adaptive control of behavior, given that only negative but not positive feedback indicates the need for change. Peterburs et al. [39] showed increased activation of cerebellar lobules VI and VIIa/Crus I for negative compared to positive feedback, and in left lobule VIIa/Crus I for the first positive feedback after switching compared to the final negative feedback before a switch. The authors pointed out that in a prior study by Lam et al. [100], no cerebellar activity was found for the feedback valence contrast likely due to the simplicity of the task itself. Therefore, task difficulty may impact how predictions are updated in the forward model [22] and could be a cause for substantial variance across the reviewed task and the respective cerebellar activation patterns. Aside from objective valence, the subjective value [33] and the timing of the feedback [137] have been shown to affect the neuronal circuits activated during learning. However, cerebellar involvement in feedback processing has not yet been investigated as a function of these factors.

### Limitations

There are several limitations to our review that need attention. Importantly, we did not include unpublished work or grey literature because we focused on peer-reviewed articles that were retrievable on PubMed following the PRISMA guidelines. Since we anticipated that it would be difficult to find appropriate studies using only this search strategy, we did consult the most relevant reviews on this topic and identified additional studies that did not report significant cerebellar findings in either the title or the abstract.

Another important limitation of this review concerns the fMRI studies. Our search strategy included the cerebellum as a key concept, among a few others, but we did not include studies that may have conducted whole brain analyses and reported no activity in the cerebellum during feedback-based learning. This is a crucial shortcoming since the chain of reasoning is solely built upon the significant cerebellar effects reported in the included studies. Nonetheless, many studies investigating feedback-based learning have focused on cortical [e.g., 138] and subcortical regions like the basal ganglia [e.g., 133, 139] and did not include the cerebellum as region of interest. However, there are also imaging studies that conducted whole-brain analyses in healthy participants and did not find or report any activation for contrasts similar to the ones described in the results section of this review [see 49, 50]. In addition, the study by Tricomi and Fiez [103] reported that the cerebellum was only partially scanned and thus further activations in the cerebellum might have remained undetected. Nevertheless, our search strategy revealed a large number of studies that reported cerebellar effects which could provide starting points for future studies.

To minimize the risk of bias, we used our screening tool and extracted data of studies that survived our inclusion criteria irrespective of the sample size and statistical method. However, the reported effects were mainly based on small sample sizes, especially in the patient populations, and therefore may have possibly limited statistical power (see supplement Table S7). In addition, the lack of studies using EEG, resting state and task-based fMRI as well as studies using non-invasive brain stimulation to stimulate the cerebellum to investigate cognitive feedback-based learning is an issue, and such results are clearly needed to complete the picture of cerebellar involvement in processing of and learning from external feedback.

Last, only studies published prior to July 2021 were included in the preregistration. However, since the peer review and publication process has taken more than 1 year, several new studies have become available. To address this limitation, we will include a brief summary of recent findings and development pertaining to the topic of this review in the following section.

### Recent Developments

In a very recent activation likelihood estimation meta-analysis on the cerebellum’s role in reward anticipation and outcome processing, Kruithof et al. [140] found bilateral activation patterns in the anterior lobe, lobule VI, left Crus I and posterior vermis across 31 studies using monetary-incentive delay tasks. In addition, activations were observed in the left lobule VI and the declive (vermian lobule VI) during processing of reward outcomes in 16 tasks. These results overlap with and complement the presently reviewed imaging findings. In a recent original study, Nicholas et al. [141] used a probabilistic feedback-based learning task and a semantic memory task in patients with cerebellar ataxia to investigate reinforcement learning in terms of prediction and prediction errors. Patients were impaired at reward learning from trial-and-error feedback but showed a preserved ability to learn to predict reward based on episodic memory. Regarding effects of cerebellar TMS on performance monitoring, a recent study reported a reduction of the ERN [142]. Due to the functional link between ERN and FRN [61], these findings certainly motivate investigations of the effects of cerebellar TMS on feedback processing as indexed by the FRN.

### Conclusions

Findings concerning the notion of altered learning from external feedback in a non-motor context in patients with cerebellar diseases are inconsistent, with roughly half of the patients showing alterations when compared to healthy controls or normative performance. This could be attributed to heterogeneity, e.g., time elapsed since lesion onset, age at lesion onset, type and location of cerebellar damage, but also small sample sizes. In contrast, degenerative diseases of the cerebellum are associated with more pronounced performance deficits compared to chronic focal lesions, although data in this regard were limited [80, 81]. Electrophysiological or imaging data in patients on the role of the cerebellum in feedback processing is extremely sparse but points to cerebellar damage being associated with altered coding of feedback valence and prediction errors [51]. Imaging data in healthy subjects yielded a much more uniform picture, with cerebellar activations found in different regions depending on task type and respective contrast. Contrasts that specifically examined feedback anticipation or feedback receipt indicated that posterolateral regions of the cerebellum play a key role in performance monitoring [e.g., 39, 73, 98, 104, 105]. However, it must be noted that a number of imaging studies in healthy subjects failed to find cerebellar activations during feedback learning [49, 50], and fMRI data on feedback learning in cerebellar patients are missing to date. Therefore, the results of this systematic review must be interpreted with caution.

Notwithstanding, we believe that performance monitoring is a relevant concept for understanding the interplay between cerebral and cerebellar structures [36], and that this concept fits well into the proposed forward model [22, 24]. Future studies therefore should not underestimate the contributions of the cerebellum to higher cognitive functions, and researchers should consider including the cerebellum as a region of interest when conducting imaging studies on feedback-based learning. We also highlight the need for more studies that use either electrophysiological measures or neuroimaging in patients with cerebellar diseases in order to better characterize the contributions of the cerebellum to processing of and learning from external feedback. It is conceivable that some of the typical deficits that patients with CCAS [15] present with, e.g., impaired verbal fluency, working memory, or affect regulation, may be at least partially rooted in aberrant processing of and learning from feedback, given that feedback processing is a critical step for generating predictions, and predictions, in turn, are helpful not only in working memory [e.g., 143], but also in social interactions [e.g., 144, for a review on the cerebellum and prediction for social contexts, see 145]. Therefore, a more comprehensive understanding of cerebellar contributions to executive functions such as performance monitoring, can help to establish and optimize treatment options for patients with diverse cerebellar diseases.

### Supplementary Information

Below is the link to the electronic supplementary material.

Supplementary file1 (DOCX 115 KB)

## Data Availability

No datasets were generated or analysed during the current study.
