# Supplementary material for: The Role of the Human Cerebellum for Learning from and Processing of External Feedback in Non-Motor Learning: A Systematic Review
Source: Cerebellum. 2024 Feb 20;23(4):1532–51. doi: 10.1007/s12311-024-01669-y (PMC11269477; doi:10.1007/s12311-024-01669-y)
Supplement: Supplementary file 1 — Supplementary file1 (DOCX 115 KB) [file 12311_2024_1669_MOESM1_ESM.docx]

**Supplemental Material: The role of the human cerebellum for learning from and processing of external feedback in non-motor learning: a systematic review**

Adam M. Berlijn^1,2,4^, Dana M. Huvermann^1,5^, Sandra Schneider^1^, Christian Bellebaum^1^,

Dagmar Timmann^5^, Martina Minnerop^2,3,4^, and Jutta Peterburs^1,6^

^1^Faculty of Mathematics and Natural Sciences, Heinrich Heine University Düsseldorf, Düsseldorf, Germany

^2^Institute of Clinical Neuroscience and Medical Psychology, Medical Faculty & University Hospital Düsseldorf, Heinrich Heine University Düsseldorf, Düsseldorf, Germany

^3^Department of Neurology, Center for Movement Disorders and Neuromodulation, Medical Faculty & Heinrich Heine University Düsseldorf, Düsseldorf, Germany

^4^Institute of Neuroscience and Medicine (INM-1), Research Centre Jülich, Jülich, Germany

^5^Department of Neurology and Center for Translational and Behavioral Neurosciences (C-TNBS), Essen University Hospital, University of Duisburg-Essen, Essen, Germany

^6^Institute of Systems Medicine and Department of Human Medicine, MSH Medical School Hamburg, Hamburg, Germany

^1^Corresponding author: berlijn@uni-duesseldorf.de

+49 211 81-14799

Department of Biological Psychology

Faculty of Mathematics and Natural Sciences

Heinrich-Heine-University Düsseldorf

Universitätsstraße 1, 40225 Düsseldorf, Germany

Key words: cerebellum, cerebellar ataxia, cognitive performance monitoring, cognitive feedback processing, reinforcement learning

Table S1 Screening tool – Abstract

| **#** | **Question** | **Yes/unsure -continue** | **No - exclude** |
| --- | --- | --- | --- |
| **1** | Does the **title or abstract** use English? |  |  |
| **2** | Does the **title or abstract** NOT indicate that a (systematic) review and/or meta-analysis was conducted? |  |  |
|  | Sample Characteristics |  |  |
| **3** | Does the **abstract** indicate that the study includes human participants? |  |  |
| **4** | Does the **abstract** indicate that the study includes a sample of healthy subjects or cerebellar patients? |  |  |
| **5** | Does the **abstract** indicate that the study includes adults? |  |  |
|  | Study Characteristics |  |  |
| **6** | Does the **abstract** indicate that the study used a quantitative design?  Keywords: mean, standard, deviation, variance, regression, covariate, modeling, ANOVA, mixed-linear model, contrast, etc. |  |  |
| **7** | Does the **abstract** indicate that the paper describes a primary study reporting original results? |  |  |
| **8** | Does the **abstract** indicate that performance monitoring was studied?  Keywords:  Cerebellum, Cerebellar Ataxia, Cerebellar, Cerebellar hemispheres performance monitoring, action monitoring, adaptive behavior, rule retrieval, executive functions Feedback Psychological, Formative Feedback, feedback, Feedback processing, reinforcement learning, prediction error, reward-based learning, associative learning, reversal learning |  |  |
| **9** | Does the **abstract** include any of the following outcomes?  e.g.: - feedback-related brain activity (e.g., FRN) or other ERP components like P300 - (choice) accuracy - learning rate  fMRI outcomes - whole brain analysis - Regions of interest (ROI) analysis (cerebellum) |  |  |
| **10** | Multiple reasons |  |  |

Table S2 Screening tool – Full-text

| **#** | **Question** | **Yes/unsure -continue** | **No - exclude** |
| --- | --- | --- | --- |
| **1** | Does the **paper** use English? |  |  |
| **2** | Does the **paper** NOT indicate that a (systematic) review and/or meta-analysis was conducted? |  |  |
|  | Sample Characteristics |  |  |
| **3** | Does the **paper** indicate that the study includes human participants? |  |  |
| **4** | Does the **paper** indicate that the study includes a sample of healthy subjects or cerebellar patients? |  |  |
| **5** | Does the **paper** indicate that the study includes a healthy and/or clinical comparison group? |  |  |
| **6** | Does the **paper** indicate that the study includes adults? |  |  |
|  | Study Characteristics |  |  |
| **7** | Does the **paper** indicate that the study used a quantitative design?  Keywords: mean, standard, deviation, variance, regression, covariate, modeling, ANOVA, mixed-linear model, contrast, etc. |  |  |
| **8** | Does the **paper** describe a primary study reporting original results? |  |  |
| **9** | Does the **paper** indicate that performance monitoring was studied?  Keywords:  Cerebellum, Cerebellar Ataxia, Cerebellar, Cerebellar hemispheres performance monitoring, action monitoring, adaptive behavior, rule retrieval, executive functions Feedback Psychological, Formative Feedback, feedback, Feedback processing, reinforcement learning, prediction error, reward-based learning, associative learning, reversal learning |  |  |
| **10** | Does the **paper** include any of the following outcomes?  e.g.: - feedback-related brain activity (e.g., FRN) or other ERPs like P300 - (choice) accuracy - learning rate - whole brain analysis - ROI analysis (cerebellum) |  |  |
| **11** | Multiple reasons |  |  |

| Table S3 Data extraction form | | | | |  |  |  |
| --- | --- | --- | --- | --- | --- | --- | --- |
| Items | Study characterization | Sample characterization | |  | |  |  |
| 1 | Article | Total sample size | | |  |  |  |
| 2 | Authors | Vascular lesion patients (*n*) | | | |  |  |
| 3 | Year | Neurodegenerative patients (*n*) | | | |  |  |
| 4 | Study type | Control (*n*) |  | |  |  |  |
| 5 | Aim of thte study | Age |  | |  |  |  |
| 6 | Results | % Female |  | |  |  |  |
| 7 | *N* Experiment | Ataxia type (if applicable) | | | |  |  |
| 8 | *N* Subjects | Control conditiond definition | | | |  |  |
| 9 | Patient study | Duration of illness in years | | | |  |  |
| 10 | Number of tasks | Handedness | | | |  |  |
| 11 | Task type | Ataxias scores | | |  |  |  |
| 12 | Task description | **Neuropsychological/ IQ Scores,** | | |  |  |  |
| 13 | Stimulus type | **Additional information** | | | |  |  |
| 14 | Feedback type |  | | |  |  |  |
| 15 | Feedback level | |  | |  |  |  |
| 16 | Behavior DVs | |  | |  |  |  |
| 17 | **Behavior Statistic** | |  | |  |  |  |
| 18 | EEG (ERP) |  |  | |  |  |  |
| 19 | EEG (Time-frequency) | |  | |  |  |  |
| 20 | Imaging (Technology) | |  | |  |  |  |
| 21 | Imaging (Whole brain) | |  | |  |  |  |
| 22 | Imaging (relevant contrast) | | | |  |  |  |
| 23 | **General results** | |  | |  |  |  |
| 24 | **Key results** |  |  | |  |  |  |
| 25 | **Conclusion** |  |  | |  |  |  |
| 26 | Addtional information | |  | |  |  |  |
| 27 | doi |  |  | |  |  |  |
| Note. Extraction categories that are highlighted in bold were added after the preregistration | | | | | | | |

| Table S4 Interrater reliability for the different screening rounds | | | | |
| --- | --- | --- | --- | --- |
|  |  |  |  |  |
| assignment | wkappa_fleiss | wkappa_CI_lower | wkappa_CI_upper | wkappa_ASE |
| test round | 1.00 | 1.00 | 1.00 | 0.00 |
| first round | 0.41 | 0.27 | 0.55 | 0.07 |
| second round | 0.50 | 0.29 | 0.72 | 0.11 |
| third round | 0.97 | 0.94 | 1.00 | 0.01 |
| Total | 0.62 | 0.53 | 0.71 | 0.05 |
|  |  |  |  |  |
| Note. Weighted Kappa (wkappa) was calculated for interrater reliability for each screening round. Lower and upper confidence interval (CI) and alpha's standard error (ASE) are provided. | | | | |

| Table S5 Overall agreement for the different screening rounds | | | | | |  |  |  |
| --- | --- | --- | --- | --- | --- | --- | --- | --- |
|  |  |  |  |  |  |  |  |  |
| assignment | n_Screened_ | n_Conflicts_ | perc_Conflicts_ | n_Inclusion_ | n_Exclusion_ | n_Maybes_ | agreement (%) |  |
| test round | 20 | 0 | 0.00 | 1 | 19 | 0 | 95.00 |  |
| first round | 300 | 48 | 16.00 | 28 | 272 | 16 | 80.00 |  |
| second round | 300 | 27 | 9.00 | 12 | 288 | 24 | 88.67 |  |
| third round | 458 | 5 | 1.09 | 21 | 437 | 5 | 94.32 |  |
| Total | 1078 | 80 | 7.42 | 62 | 1016 | 45 | 88.78 |  |
|  |  |  |  |  |  |  |  |  |
| Note. *n* = 21 articles that were not found in the PubMed database were screened in the third round. | | | | | | | |  |

| Table S6 Synthesis of studies according to the four hypotheses | |  |  |
| --- | --- | --- | --- |
|  |  |  |  |
| Hypothesis | Number of studies | Patient studies | Non-patient studies |
| 1 (Behavior) | 10 | 10 | 0 |
| 2 (Electrophysiological) | 1 | 1 | 0 |
| 3 (Imaging: Cerebellar activation pattern) | 25 | 0 | 25 |
| 4 (Non-invasive brain stimulation) | 0 | 0 | 0 |
|  |  |  |  |
| Total number of studies | 36 | 11 | 25 |
|  | | | |

| Table S7. Disease type and number of patients included into the behavioral section | | | | | |
| --- | --- | --- | --- | --- | --- |
|  |  |  |  |  |  |
| Study | Year | Type of disease | Onset of disease | *n* | Further details |
| Schmahmann & Sherman^*^ | 1998 | Cerebellar cortical atrophy | 4-6 years | 3 | NA |
|  |  | Stroke (PICA, SCA) | Weeks-months | 13 | 2 Bilateral PICA stroke 3 Right PICA stroke 1 Right PICA (medial) stroke  1 Right PICA (branch) stroke 3 Left PICA stroke 1 Right AICA stroke 1 Left SCA stroke 1 Right SCA stroke |
|  |  | Postinfectious cerebellitis | 1-3 Months | 3 | NA |
|  |  | Tumor resection | 1 week | 1 | Cerebellar vermis excision for tumor (ganglioglioma) |
|  |  |  |  |  | ^*^*n* = 8 participants took part in the WCST |
| Rustemeier et al. | 2016 | Stroke (PICA, bilateral) | 68.25 (10-238 months) | 12 | 5 Left posterior cerebellar lobe (PICA & SCA; 1 patient with disturbed blood flow in the territory of the posterior cerebral artery)  6 Right posterior cerebellar lobe (PICA)  1 Left medial dentate nucleus, right posterior/ right anterior cerebellar lobe |
| Drepper et al. | 1999 | Idiopathic cerebellar ataxia | 4.4 (*SD* +- 3) years | 9 | NA |
| Gottwald et al.^*^ | 2004 | Intracerebellar heamatomas | NA | 4 | Intracerebellar heamatomas |
|  |  | Cerebellar tumors | Years | 17 | Cerebellar tumors (5 meningeomas, 7 metastasis, 2 haemangiomas, 2 angiomas, 1 ganglioglioma)  ^*^*n* = 19 participants took part in the MCST |
| Turner et al. | 2007 | Cerebellar infarction (stroke) | 14-180 days | 6 | 1 left side damage to the lateral anterior lobe, deep into the corpus, just posterior and lateral to the dentate and may have impacted the dentate, small lesion right side of Crus II within the intermediate region/ small portion of the right vermis impacting lobules VIII and IX as well as the tonsil 1 had bilateral lesions in the medial and intermediate regions of Crus II of VIIa, VIIb, and VIII, atrophy throughout the cerebellum and cerebrum 1 damage to lobe VIII and Crus II of VIIb 1 damage to Crus I and lobe VIII 1 damage to the right cerebellum in area Crus II 1 damage to the right-half of the inferior vermis, very small part of Crus I |
| Manes et al. | 2009 | Cerebellar infarction (stroke) | 6 months | 11 | 7 posterior inferior cerebellar artery (PICA; 4 right, 1 left, 2 bilateral) 3 infarctions in the territory of the left superior cerebellar artery (SCA) 1 Infarction in both territories, PICA and SCA |
| Thoma et al. | 2008 | Selective vascular lesions of the cerebellum | 60.4 (48-73) months | 8 | 3 Unilateral right cerebellar hemisphere lesion (one with an additional lesion of the deep cerebellar nuclei) 3 Unilateral left cerebellar hemisphere lesion (one with an additional lesion of the deep cerebellar nuclei/ probably dentate  2 Bilateral lesions (one with possible damage of cerebellar nuclei) |
| Dirnberger et al. | 2010 | Isolated ischemic infarction | 6 months (0.6 – 5.5 years) | 11 | 3 Infarction in the left hemisphere 4 Infarction in the right hemisphere  4 Infarction in both hemispheres |
| Mak et al. | 2015 | Isolated cerebellar tumors (mentioned as cancer without metastases) | 6 months | 30 | 18 vermis and medial part of the Cerebellum  6 Extreme lateral parts of the cerebellar hemispheres  3 Lateral parts of the cerebellar hemispheres  3 Spread over all areas of the cerebellum |
| Mukhopadhyay et al. | 2007 | Cerebellar lesion (stroke or tumor) | NA | 10 | NA |
| Tucker et al. | 1996 | Cerebellar Ataxia | 3 years 15 years 1 years 5 years 12 years 30+ years 10 years | 7 | Autosomal dominant CA Type I - CB & brainstem atrophy (MRI) Idiopathic late onset CA (SEV:15) - Mild CB atrophy (CT) Idiopathic late onset CA - CB atrophy (CT) Autosomal dominant C A Type III (SEV:13) - CB atrophy (MRI) Idiopathic late onset CA (SEV: 15) - CB & brainstem atrophy (MRI) Early onset ataxia with retained reflexes (SEV: 14) - CB & brainstem atrophy (MRI) Autosomal recessive late onset CA (SEV: 14) - CB & cervical cord atrophy (MRI) |
|  |  |  |  |  |  |
| Total number of patients | | | | 131^1^ | |
| Note. SCA = Superior cerebellar artery, PICA = Posterior inferior cerebellar artery, AICA = Anterior inferior cerebellar artery, NA = data not available. ^1^Corrected for participants who actually took part in the respective feedback learning task. | | | | | |

| Table S8. Short description of each study included into the review (sorted by year of publication)   \| Author \| Year \| Type \| Patients \| Sample \| Aim of the study \| Paradigm \| Feedback \| Results \| Hypo. \| \| --- \| --- \| --- \| --- \| --- \| --- \| --- \| --- \| --- \| --- \| \| Berman et al. \| 1995 \| Imaging \| no \| 40 \| Neural processes underlying the WCST \| WCST + PET \| Correct, Incorrect \| High performance in WCST; Cerebellar activation during the WCST in comparison to a control task in healthy subjects. \| Image \| \| Tucker et al. \| 1996 \| Behavior \| yes \| 7 \| Investigating if cerebellar pathology causes impaired associative learning. \| Associative Learning Task \| Correct, Incorrect \| Patients with cerebellar degeneration made more errors than healthy, age and IQ matched controls. However, Huntington patients also showed this kind of impairment but Parkinson patients did not. \| Behav \| \| Nagahama et al. \| 1996 \| Imaging \| no \| 18 \| Cerebral regions involved during the Card Sorting Test. \| MCST/MTS + PET \| Correct, Incorrect \| High performance in MCST; Bilateral and left cerebellar activity for several contrasts. \| Image \| \| Schmahmann & Sherman \| 1998 \| Behavior \| yes \| 8 did the WCST (out of 20) \| Performance of patients with different cerebellar diseases in the WCST \| WCST \| Correct, Incorrect \| No overall group difference between the patients and the norm sample. However, differences are present within the patients according to the type of disease. \| Behav* \| \| Drepper et al. \| 1999 \| Behavior \| yes \| 9 \| Role of the cerebellum for cognitive associative learning \| Associative Learning Task \| Correct, Incorrect \| Patients took longer to reach learning criterion and had more difficulties in naming the correct association, no differences due to extra cerebellar lesions or sample differences. \| Behav \| \| Knutson et al. \| 2001 \| Imaging \| no \| 8 \| Neural circuity of the anticipation of monetary rewards \| Monetary Incentive Delay Task (MID) + fMRI \| Win or Loss \| Large vs. small reward and punishment anticipation as contrasts were both associated with increased activation in the cerebellar vermis. \| Image \| \| Ernst et al. \| 2002 \| Imaging \| no \| 20 \| Investigated regional brain activation associated with decision making \| Risk Taking Task (RTT), Control Task + PET \| Win or Loss \| Lateral cerebellum (bilateral, dominant left side) active during the risk-taking task. Bilateral and right cerebellar peduncle was active for the contrast learned vs. unlearned task. \| Image \| \| Knutson et al. \| 2003 \| Imaging \| no \| 12 \| Regions of the mPFC involved in tracking monetarily rewarding outcomes \| Monetary Incentive Delay Task (MIDT) + fMRI \| Win or Loss \| Anticipation of potential gain vs. no outcome and potential loss vs. no outcome were both associated with increased activation in the cerebellar vermis. \| Image \| \| Tanaka et al. \| 2004 \| Imaging \| no \| 20 \| Investigated the neural pathways for reward prediction at different time scales \| Markov decision task + fMRI \| Monetary (3 small and 3 large rewards/losses) \| Medial cerebellum active during immediate reward prediction. Lateral cerebellum active in future rewards predictions. Cerebellum active during early stage of learning. \| Image \| \| Gottwald et al. \| 2004 \| Behavior \| yes \| 39: 19 (21) patients, 20 (21) controls \| Investigation if (undamaged) cerebellum necessary for higher cognitive processes \| MCST \| Correct or Incorrect \| No significant difference between patients and controls for performance in the MCST and the categories when left to right hemisphere lesions were compared to each other. \| Behav* \| \| Remijnse et al. \| 2005 \| Imaging \| no \| 27 \| Neural circuity of reversal learning \| Probabilistic reversal learning task + fMRI \| Correct or Incorrect \| Left cerebellar activation patterns when reward and punishment feedback were mediated as could be seen in the dissociation of stimulus – punishment association and affective switching. \| Image \| \| Lie et al. \| 2006 \| Imaging \| no \| 12 \| Neural processes underlying the WCST \| WCST + fMRI \| Correct or Incorrect \| Cerebellum is involved in matching information (bilateral cerebellum), working memory (right cerebellum), error detection and feedback processing (bilateral cerebellum). Cerebellum active for the contrast instructed vs. uninstructed set shifting. \| Image \| \| Bjork & Hommer \| 2007 \| Imaging \| no \| 20 \| Investigation on the dependency of the ventromedial striatum activation on the interaction between reward delivery probabilities and instrumental response \| Factorial Reward Anticipation (FRA) task (a variant of the MID task) + fMRI \| Win or Loss \| Cerebellum involved in the anticipatory process of encoding different reward probabilities, the requirement to execute a motor response and the processing of feedback type. \| Image \| \| Marco-Pallarés et al. \| 2007 \| Imaging \| no \| 12 \| Define the neural correlates of feedback processing \| Associative Learning Task + fMRI \| Correct or Incorrect \| Significant increase in the activity in the right cerebellum for positive feedback compared with negative feedback trials. \| Image \| \| Mukhopadhyay et al. \| 2007 \| Behavior \| yes \| 20: 10 patients, 10 healthy controls \| Neuroanatomical processes underlying the WCST (set-shifting ability) \| WCST \| Correct or Incorrect \| Performance of patients with cerebellar lesions showed significant less numbers of categories completed and perseverative errors. No significant difference in the percentage of perseverative responses. Cortical and subcortical damages had a stronger impact on performance than cerebellar lesions. \| Behav \| \| Turner et al. \| 2007 \| Behavior \| yes \| 15: 6 patients, 9 healthy controls \| Investigation on the role of the cerebellum in emotion \| WCST \| Correct or Incorrect \| No significant difference between stroke patients with a cerebellar lesion and healthy controls for the WCST. \| Behav* \| \| Tricomi & Fiez \| 2008 \| Imaging \| no \| 11 \| Investigated the extent to which the context of feedback information differentially affected processing in the ncl. caudatus. \| Feedback-based paired association word-learning task + fMRI \| Positive Negative \| More activation for incorrect than correct trials was found in the left medial inferior cerebellum when arbitrary associations have been relearned through feedback and these associations have been tested by feedback reflecting the correctness of the memory in the last round (round × accuracy × time interaction). Bilateral medial inferior cerebellum and anterior cerebellum were stronger activated for incorrect than correct trials in an accuracy time interaction. \| Image \| \| Thoma et al. \| 2008 \| Behavior \| yes \| 32: 8 patients, 24 healthy controls \| Role of cerebellar focal lesions for different associative learning tasks \| Two Probabilistic Learning Tasks + MCST \| Reward (+5 vs. +20cent) vs. No reward \| Small cerebellar lesions impaired reward-based reversal learning in the probabilistic learning tasks, however, no differences in performance were found for the MCST. \| Behav* \| \| Bischoff-Grethe et al. \| 2009 \| Imaging \| no \| 12 \| Identification of brain regions that were differentially responsive to positive and negative feedback as well as areas that were responsive to both types of informative feedback \| Associative Learning Task + fMRI \| Correct, Incorrect, unknown \| Right cerebellar lobule VI was activated following both positive and negative feedback. \| Image \| \| Manes et al. \| 2009 \| Behavior \| yes \| 22: 11 patients, 11 healthy controls \| Investigated executive functions in patients with cerebellar lesions \| modified version of the WCST \| Correct or Incorrect \| Significant differences between patients with focal vascular lesions and healthy controls on the performance in the WCST. \| Behav \| \| Dirnberger et al. \| 2010 \| Behavior \| yes \| 24: 11 patients, 13 healthy controls \| Performance in a visuomotor task that is interfered by a modified WCST in patients with cerebellar stroke \| modified version of the WCST \| Correct or Incorrect \| No differences between patients and controls regarding the performance in the modified WCST. \| Behav* \| \| Linke et al. \| 2010 \| Imaging \| no \| 33 \| Investigated how extrinsic and intrinsic motivation modulates the neural response to reward and punishment as well as learning from reward and punishment \| Probabilistic reversal learning task + fMRI \| Correct, Incorrect, (neutral: "Choice made") \| Activation in the left cerebellum was found during behavioral switching for the contrast final reversal error minus baseline. \| Image \| \| Balsters & Ramnani \| 2011 \| Imaging \| no \| 19 \| Investigated if the cerebellum is involved in learning low-order rules which lead to automatized behavior \| Dual Task + fMRI \| Relevant (Correct: green dot, Incorrect: red dot), Miss (yellow dot) \| Rule information was manipulated, and cerebellar excitability changes were found as activation in Crus I for high order cues that had decreased faster than for low order cues. Results support the internal model concept. \| Image \| \| Greening et al. \| 2011 \| Imaging \| no \| 21 \| Investigated the neural processes of reversal learning. Reversal learning was distinguished into inhibition of response and overcoming avoidance as subprocess \| Object discrimination reversal learning task + fMRI \| Positive (You WIN 100 points!)/Negative feedback (You LOSE 100 points!) \| Early attempts in the task to overcome response inhibition were associated with activation in the cerebellum. \| Image \| \| Bellebaum et al. \| 2012 \| Imaging \| no \| 30 \| Investigated the effect of expectancy during active and observational learning \| Probabilistic Learning + fMRI \| Positive reward (+20cent) vs. no reward (0cent) vs. negative reward (-20cent) \| Cerebellar activation was found for different expectancy manipulations and prediction errors in active and observational learners and between both groups. \| Image \| \| Balsters et al. \| 2013 \| Imaging \| no \| 15 \| Influence of abstract information as low and high-order rules on prefrontal projecting cerebellar lobules \| Delayed-response Task + fMRI \| Correct (green dot) or Incorrect (red dot) \| Prefrontal-projecting cerebellar lobules Crus I and II were both active during the processing of both first- and second order rules. Cerebellar activation was not driven by motor activation. \| Image \| \| Lam et al. \| 2013 \| Imaging \| no \| 21 \| Neuronal processing of implicit learning and the impact of content and feedback on it \| Weather prediction task + fMRI \| Smiley or Frowney \| Activation of the lateral cerebellar hemisphere due to higher predictive values. More activation of the right lateral cerebellum in high-value trials compared to low-value trials. Results support the internal model concept. \| Image \| \| Späti et al. \| 2014 \| Imaging \| no \| 25 \| Influence of agency and valence on the processing of feedback \| modified version of the Dynamically adapted motion prediction task (DAMP) + fMRI \| Win (+50) or Loss (-50) \| Cerebellar vermis active in the contrast self-attributed (SA) vs. externally attributed (EA). Cerebellar activation was not driven by motor activation. \| Image \| \| Kobza & Bellebaum \| 2015 \| Imaging \| no \| 31 \| Neural processes of active vs. observed learning from feedback. \| Probabilistic learning card-guessing paradigm + fMRI \| Win (green +50 cent) or Loss (red -50 cent) \| Cerebellar vermis active for unexpected rewards. Lateral cerebellum active for highly predictive cues that appeared before the outcome. Cerebellar activation was more pronounced for action-dependent prediction errors in the active compared to the observer sample. Cerebellar prediction error is suggested to be independent from the reward system. \| Image \| \| Von der Gablentz et al. \| 2015 \| Imaging \| no \| 16 \| Investigated the neural processes underlying task-switching and performance monitoring. \| modified version of the Eriksen-Flanker Task + fMRI \| Correct (green square) or Incorrect (red square) \| Cerebellar vermis active for the contrast switch feedback (switch) vs. correct feedback. The contrast error feedback and switch feedback revealed activation in Cerebellar Crus I and II. Cerebellum involved in switch and performance feedback. \| Image \| \| Mak et al. \| 2016 \| Behavior \| yes \| 60: 30 patients, 30 healthy controls \| Investigated executive functions in a sample of patients after cerebellar surgery. \| WCST \| Correct or Incorrect \| Patients made more errors and perseverative errors, gave more perseverative responses, and had a lower number of categories completed in comparison to healthy controls. \| Behav \| \| Rustemeier et al. \| 2016 \| EEG \| yes \| 37: 12 patients, 25 healthy controls \| Investigated learning from positive and negative feedback in a sample with patients with different cerebellar lesions. \| Probabilistic feedback learning task + EEG \| Positive (+50cent) or Negative (-20 cent) \| Performance of the patients did not differ in comparison to healthy controls. ERP components FRN and P300 were altered in patients and showed higher difference signal amplitudes. Goes along with altered processing of positive and negative feedback. \| EEG, Behav* \| \| Shao et al. \| 2016 \| Imaging \| no \| 26 \| Investigated the influence of illusion of control and gambler’s fallacy in a decision-making task. \| Card-guessing game + fMRI \| Win ("YOU WON! :)”) or Loss ("YOU LOST :(") \| Activation of the bilateral cerebellum during betting for the contrast Pre-win > Pre-loss. Cerebellum involved in the process of decision making and affected by previous decision (win vs. loss). Suggested to differently update the generated internal model for predicting a good bet. \| Image \| \| Peterburs et al. \| 2018 \| Imaging \| no \| 31 \| Role of the cerebellum in reversal learning by determining if the cerebellum differentially processes feedback as a function of changes in response strategy in a volatile environment. \| Reversal learning task + fMRI \| Win (+10 cent) or Loss (-10 Cent) \| Increased activation in cerebellar lobule VI and VIIa/Crus I in the first contrast (negative relative to positive feedback). The contrast first positive feedback > final negative feedback and final negative feedback > negative stay feedback was associated with increased activation in posterolateral regions of the cerebellum (lobule VI/VIIa/Crus I/Crus II). \| Image \| \| Edde et al. \| 2019 \| Imaging \| no \| 31: 17 young group: median age 24 years ± IQR 6; 14 old group median age 67 years ± IQR 4 \| Investigated how a visuomotor learning task would modify cerebellar intrinsic connectivity in groups of young and older male subjects. \| Conditional visuomotor task + fMRI \| green, yellow or red circle \| Second level analysis with the contrast post-task rest > pre-task-rest. Young subjects: post-learning changes of functional connectivity within cerebellar networks (cerebello-frontal, cerebello-temporal, and cerebello-cerebellar connections). Functional connectivity associated with task performance for several cerebello-cortical connections. Older subjects: Less changes in the activity for the resting state functional connectivity than in young subjects. Did not involve cerebellar network. \| Image \| \| Jackson et al. \| 2020 \| Imaging \| no \| 50: 26 young adults: mean age = 22.54 ± 2.87; age range = 18–30; 24 Old adults: mean age = 72.24 ± 6.29; age range = 60–84; \| Investigated age related differences in task performance and functional activation patterns in older adults and younger adults. \| Second-order rule learning task + fMRI \| Correct (green circle), Incorrect (red circle) or Missed (in red) \| Local peak activation in the right lobule VI for the instruction cues in the learning blocks in the sample with older adults. Cluster included activation in Crus I and II regions but no local peak activation. Within young adults, bilateral Crus II, right Crus I, right lobule VI, and right lobule VIII were activated. Older adults showed more widespread activation compared to young adults. Left Crus II and lobule VI activation in older adults for the feedback cues in learning blocks as well as activation in lobule III. Cerebellum involved in error-related cognitive processes. \| Image \| |  |
| --- | --- | --- | --- | --- | --- | --- | --- | --- | --- | --- | --- | --- | --- | --- | --- | --- | --- | --- | --- | --- | --- | --- | --- | --- | --- | --- | --- | --- | --- | --- | --- | --- | --- | --- | --- | --- | --- | --- | --- | --- | --- | --- | --- | --- | --- | --- | --- | --- | --- | --- | --- | --- | --- | --- | --- | --- | --- | --- | --- | --- | --- | --- | --- | --- | --- | --- | --- | --- | --- | --- | --- | --- | --- | --- | --- | --- | --- | --- | --- | --- | --- | --- | --- | --- | --- | --- | --- | --- | --- | --- | --- | --- | --- | --- | --- | --- | --- | --- | --- | --- | --- | --- | --- | --- | --- | --- | --- | --- | --- | --- | --- | --- | --- | --- | --- | --- | --- | --- | --- | --- | --- | --- | --- | --- | --- | --- | --- | --- | --- | --- | --- | --- | --- | --- | --- | --- | --- | --- | --- | --- | --- | --- | --- | --- | --- | --- | --- | --- | --- | --- | --- | --- | --- | --- | --- | --- | --- | --- | --- | --- | --- | --- | --- | --- | --- | --- | --- | --- | --- | --- | --- | --- | --- | --- | --- | --- | --- | --- | --- | --- | --- | --- | --- | --- | --- | --- | --- | --- | --- | --- | --- | --- | --- | --- | --- | --- | --- | --- | --- | --- | --- | --- | --- | --- | --- | --- | --- | --- | --- | --- | --- | --- | --- | --- | --- | --- | --- | --- | --- | --- | --- | --- | --- | --- | --- | --- | --- | --- | --- | --- | --- | --- | --- | --- | --- | --- | --- | --- | --- | --- | --- | --- | --- | --- | --- | --- | --- | --- | --- | --- | --- | --- | --- | --- | --- | --- | --- | --- | --- | --- | --- | --- | --- | --- | --- | --- | --- | --- | --- | --- | --- | --- | --- | --- | --- | --- | --- | --- | --- | --- | --- | --- | --- | --- | --- | --- | --- | --- | --- | --- | --- | --- | --- | --- | --- | --- | --- | --- | --- | --- | --- | --- | --- | --- | --- | --- | --- | --- | --- | --- | --- | --- | --- | --- | --- | --- | --- | --- | --- | --- | --- | --- | --- | --- | --- | --- | --- | --- | --- | --- | --- | --- | --- | --- | --- | --- | --- | --- | --- | --- | --- | --- | --- | --- | --- | --- | --- | --- | --- | --- | --- | --- | --- | --- | --- | --- | --- | --- | --- | --- | --- | --- | --- | --- | --- | --- | --- | --- | --- | --- | --- |

Note. Studies were sorted according to the year of publication. Hypo. = Hypotheses. Hypothesis 1 = Behavior (Behav), Hypothesis 2 = Electrophysiological (EEG), Hypothesis 3 = Imaging (Image).

| Table S9 Coordinates of all significant cerebellar activations in MNI space | | | | | | | | | | | | | | | | | | | | | | | | | | | | | | | | | | | | | | | |
| --- | --- | --- | --- | --- | --- | --- | --- | --- | --- | --- | --- | --- | --- | --- | --- | --- | --- | --- | --- | --- | --- | --- | --- | --- | --- | --- | --- | --- | --- | --- | --- | --- | --- | --- | --- | --- | --- | --- | --- |
|  | |  | | | | | | | | |  | | | | | | | | |  | | | | | | | | |  | | | | | | | | |  | |
| **Berman et al., 1995** | | | | | | | | | | |  | | | | | | | | |  | | | | | | | | |  | | | | | | | | |  | |
|  | |  | | | | | | | | |  | | | | | | | | |  | | | | | | | | |  | | | | | | | | |  | |
| Statistical parametric mapping: Foci of maximal activation (WCST>control task) | | | | | | | | | | | | | | | | | | | | | | | | | | | | | | | | | | | | | | | |
| Coordinates | | | | | | | | | | | | | | | | | | | | Region | | | | | | | | | Side | | | | | | | | |  | |
| x | | y | | | | | | | | | z | | | | | | | | | MNI | | | | | | | | |  | | | | | | | | |  | |
| 29 | | -66 | | | | | | | | | -24 | | | | | | | | | Cerebellum (Lobule VI) | | | | | | | | | Right | | | | | | | | |  | |
| -22 | | -81 | | | | | | | | | -24 | | | | | | | | | Cerebellum (Crus I) | | | | | | | | | Left | | | | | | | | |  | |
| -17 | | -77 | | | | | | | | | -24 | | | | | | | | | Cerebellum (Crus I) | | | | | | | | | Left | | | | | | | | |  | |
| 24 | | -54 | | | | | | | | | -24 | | | | | | | | | Cerebellum (Lobule VI) | | | | | | | | | Right | | | | | | | | |  | |
|  | |  | | | | | | | | |  | | | | | | | | |  | | | | | | | | |  | | | | | | | | |  | |
| **Nagahama et al. 1996** | | | | | | | | | | |  | | | | | | | | |  | | | | | | | | |  | | | | | | | | |  | |
|  | |  | | | | | | | | |  | | | | | | | | |  | | | | | | | | |  | | | | | | | | |  | |
| Activated cerebral areas during the MTS task with selective attention to each category | | | | | | | | | | | | | | | | | | | | | | | | | | | | | | | | | | | | | | | |
|  | |  | | | | | | | | |  | | | | | | | | |  | | | | | | | | |  | | | | | | | | |  | |
| Number | |  | | | | | | | | |  | | | | | | | | |  | | | | | | | | |  | | | | | | | | |  | |
| Coordinates | | | | | | | | | | | | | | | | | | | | Region | | | | | | | | | Side | | | | | | | | |  | |
| x | | y | | | | | | | | | z | | | | | | | | | MNI | | | | | | | | |  |  |  |  |  |  |  |  |  |  | |
| 18 | | -49 | | | | | | | | | -38 | | | | | | | | | Cerebellum (Lobule VIII) | | | | | | | | | Right | | | | | | | | |  | |
| 28 | | -57 | | | | | | | | | -38 | | | | | | | | | Cerebellum (Crus I) | | | | | | | | | Right | | | | | | | | |  | |
| 47 | | -68 | | | | | | | | | -38 | | | | | | | | | Cerebellum (Crus II) | | | | | | | | | Right | | | | | | | | |  | |
| 10 | | -55 | | | | | | | | | -33 | | | | | | | | | Cerebellum (Lobule IX) | | | | | | | | | Right | | | | | | | | |  | |
|  | |  | | | | | | | | |  | | | | | | | | |  | | | | | | | | |  | | | | | | | | |  | |
| Colour | |  | | | | | | | | |  | | | | | | | | |  | | | | | | | | |  | | | | | | | | |  | |
| Coordinates | | | | | | | | | | | | | | | | | | | | Region | | | | | | | | | Side | | | | | | | | |  | |
| x | | y | | | | | | | | | z | | | | | | | | | MNI | | | | | | | | |  |  |  |  |  |  |  |  |  |  | |
| 26 | | -59 | | | | | | | | | -33 | | | | | | | | | Cerebellum (Lobule VI) | | | | | | | | | Right | | | | | | | | |  | |
| 35 | | -68 | | | | | | | | | -24 | | | | | | | | | Cerebellum (Lobule VI) | | | | | | | | | Right | | | | | | | | |  | |
| 34 | | -59 | | | | | | | | | -29 | | | | | | | | | Cerebellum (Lobule VI) | | | | | | | | | Right | | | | | | | | |  | |
|  | |  | | | | | | | | |  | | | | | | | | |  | | | | | | | | |  | | | | | | | | |  | |
| Shape | |  | | | | | | | | |  | | | | | | | | |  | | | | | | | | |  | | | | | | | | |  | |
| Coordinates | | | | | | | | | | | | | | | | | | | | Region | | | | | | | | | Side | | | | | | | | |  | |
| x | | y | | | | | | | | | z | | | | | | | | | MNI | | | | | | | | |  |  |  |  |  |  |  |  |  |  | |
| -31 | | -61 | | | | | | | | | -33 | | | | | | | | | Cerebellum (Crus I) | | | | | | | | | Left | | | | | | | | |  | |
| -25 | | -69 | | | | | | | | | -37 | | | | | | | | | Cerebellum (Crus II) | | | | | | | | | Left | | | | | | | | |  | |
| 19 | | -74 | | | | | | | | | -34 | | | | | | | | | Cerebellum (Crus I) | | | | | | | | | Right | | | | | | | | |  | |
| 24 | | -56 | | | | | | | | | -24 | | | | | | | | | Cerebellum (Lobule VI) | | | | | | | | | Right | | | | | | | | |  | |
| 10 | | -71 | | | | | | | | | -29 | | | | | | | | | Cerebellum (Crus I) | | | | | | | | | Right | | | | | | | | |  | |
| -1 | | -71 | | | | | | | | | -34 | | | | | | | | | Cerebellum Vermis VIII | | | | | | | | | Left | | | | | | | | |  | |
|  | |  | | | | | | | | |  | | | | | | | | |  | | | | | | | | |  | | | | | | | | |  | |
| Activated cerebral areas in the composite for all the MTS tasks compared with those in the resting condition | | | | | | | | | | | | | | | | | | | | | | | | | | | | | | | | | | | | | | | |
| Coordinates | | | | | | | | | | | | | | | | | | | | | Region | | | | | | | | Side | | | | | | | |  | | |
| x | | | y | | | | | | | | | z | | | | | | | | | MNI | | | | | | | |  |  |  |  |  |  |  |  |  | | |
| 22 | | | -51 | | | | | | | | | -38 | | | | | | | | | Cerebellum (Lobule VIII) | | | | | | | | Right | | | | | | | |  | | |
| 32 | | | -60 | | | | | | | | | -24 | | | | | | | | | Cerebellum (Lobule VI) | | | | | | | | Right | | | | | | | |  | | |
| 4 | | | -71 | | | | | | | | | -29 | | | | | | | | | Cerebellum (Vermis VII) | | | | | | | | Right (Region V) | | | | | | | |  | | |
| -46 | | | -58 | | | | | | | | | -37 | | | | | | | | | Cerebellum (Crus I) | | | | | | | | Left | | | | | | | |  | | |
| -35 | | | -52 | | | | | | | | | -37 | | | | | | | | | Cerebellum (Crus I) | | | | | | | | Left | | | | | | | |  | | |
|  | | |  | | | | | | | | |  | | | | | | | | |  | | | | | | | |  | | | | | | | |  | | |
| Activated cerebral areas during the MCST compared with each MTS task | | | | | | | | | | | | | | | | | | | | | | | | | | | | | | | | | | | | | | | |
|  | | |  | | | | | | | | |  | | | | | | | | |  | | | | | | | |  | | | | | | | |  | | |
| MCST versus number-matching task | | | | | | | | | | | | | | | | | | | | |  | | | | | | | |  | | | | | | | |  | | |
| Coordinates | | | | | | | | | | | | | | | | | | | | | Region | | | | | | | | Side | | | | | | | |  | | |
| x | | | y | | | | | | | | | z | | | | | | | | | MNI | | | | | | | |  |  |  |  |  |  |  |  |  | | |
| -15 | | | -66 | | | | | | | | | -33 | | | | | | | | | Cerebellum (Lobule VIII) | | | | | | | | Left | | | | | | | |  | | |
| -37 | | | -71 | | | | | | | | | -33 | | | | | | | | | Cerebellum (Crus I) | | | | | | | | Left | | | | | | | |  | | |
| -21 | | | -72 | | | | | | | | | -38 | | | | | | | | | Cerebellum (Crus II) | | | | | | | | Left | | | | | | | |  | | |
|  | | |  | | | | | | | | |  | | | | | | | | |  | | | | | | | |  | | | | | | | |  | | |
| MCST versus colour-matching task | | | | | | | | | | | | | | | | | | | | |  | | | | | | | |  | | | | | | | |  | | |
| Coordinates | | | | | | | | | | | | | | | | | | | | | Region | | | | | | | | Side | | | | | | | |  | | |
| x | | | y | | | | | | | | | z | | | | | | | | | MNI | | | | | | | |  |  |  |  |  |  |  |  |  | | |
| -23 | | | -79 | | | | | | | | | -29 | | | | | | | | | Cerebellum (Crus I) | | | | | | | | Left | | | | | | | |  | | |
|  | | |  | | | | | | | | |  | | | | | | | | |  | | | | | | | |  | | | | | | | |  | | |
| MCST versus shape-matching task | | | | | | | | | | | | | | | | | | | | |  | | | | | | | |  | | | | | | | |  | | |
| Coordinates | | | | | | | | | | | | | | | | | | | | | Region | | | | | | | | Side | | | | | | | |  | | |
| x | | | y | | | | | | | | | z | | | | | | | | | MNI | | | | | | | |  |  |  |  |  |  |  |  |  | | |
| -11 | | | -81 | | | | | | | | | -38 | | | | | | | | | Cerebellum (Crus II) | | | | | | | | Left | | | | | | | |  | | |
| -20 | | | -85 | | | | | | | | | -34 | | | | | | | | | Cerebellum (Crus II) | | | | | | | | Left | | | | | | | |  | | |
|  | | |  | | | | | | | | |  | | | | | | | | |  | | | | | | | |  | | | | | | | |  | | |
| Activated cerebral areas during the MCST compared with those during the composite for all the matching-to sample tasks | | | | | | | | | | | | | | | | | | | | | | | | | | | | | | | | | | | | | | | |
| Coordinates | | | | | | | | | | | | | | | | | | | | | Region | | | | | | | | Side | | | | | | | |  | | |
| x | | | y | | | | | | | | | z | | | | | | | | | MNI | | | | | | | |  |  |  |  |  |  |  |  |  | | |
| -19 | | | -78 | | | | | | | | | -38 | | | | | | | | | Cerebellum (Crus II) | | | | | | | | Left | | | | | | | |  | | |
| -39 | | | -67 | | | | | | | | | -28 | | | | | | | | | Cerebellum (Crus I) | | | | | | | | Left | | | | | | | |  | | |
| 25 | | | -80 | | | | | | | | | -38 | | | | | | | | | Cerebellum (Crus II) | | | | | | | | Right | | | | | | | |  | | |
| 31 | | | -71 | | | | | | | | | -38 | | | | | | | | | Cerebellum (Crus II) | | | | | | | | Right | | | | | | | |  | | |
|  | | |  | | | | | | | | |  | | | | | | | | |  | | | | | | | |  | | | | | | | |  | | |
| **Knutson et al. 2001** | | | | | | | | | | | | | | | |  | | | | | | | |  | | |  | | | | | | | | |  | | | |
|  | | | | | | |  | | | | | | | | |  | | | | | | | |  | | |  | | | | | | | | |  | | | |
| Large versus small reward anticipation | | | | | | | | | | | | | | | | | | | | | | | | | | | | | | | | | | | |  | | | |
| Coordinates | | | | | | | | | | | | | | | | | | | | | | | | Region | | | Side | | | | | | | | |  | | | |
| x | | | | | | | y | | | | | | | | | z | | | | | | | | MNI | | |  |  |  |  |  |  |  |  |  |  | | | |
| -1 | | | | | | | -49 | | | | | | | | | -17 | | | | | | | | Cerebellum Vermis (IV, V) | | | Left | | | | | | | | |  | | | |
|  | | | | | | |  | | | | | | | | |  | | | | | | | |  | | |  | | | | | | | | |  | | | |
| Large versus small punishment anticipation | | | | | | | | | | | | | | | | | | | | | | | | | | |  | | | | | | | | |  | | | |
| Coordinates | | | | | | | | | | | | | | | | | | | | | | | | Region | | | Side | | | | | | | | |  | | | |
| x | | | | | | | y | | | | | | | | | z | | | | | | | | MNI | | |  |  |  |  |  |  |  |  |  |  | | | |
| 5 | | | | | | | -61 | | | | | | | | | -39 | | | | | | | | Cerebellum Vermis (IX) | | | Right | | | | | | | | |  | | | |
|  | | | | | | |  | | | | | | | | |  | | | | | | | |  | | |  | | | | | | | | |  | | | |
| **Ernst et al. 2002** | | | | | | | | | | | | | | | |  | | | | | | | |  | | |  | | | | | | | | |  | | | |
|  | | | | | | |  | | | | | | | | |  | | | | | | | |  | | |  | | | | | | | | |  | | | |
| Statistical Parametric Mapping Activation (Active Task - Control Task) | | | | | | | | | | | | | | | | | | | | | | | | | | | | | | | | | | | | | | | |
| Coordinates | | | | | | | | | | | | | | | | | | | | | | | | Region | | | Side | | | | | | | | |  | | | |
| x | | | | | | | y | | | | | | | | | z | | | | | | | | MNI | | |  |  |  |  |  |  |  |  |  |  | | | |
| 47 | | | | | | | -62 | | | | | | | | | -38 | | | | | | | | Cerebellum (Crus I) | | | Right | | | | | | | | |  | | | |
| -41 | | | | | | | -71 | | | | | | | | | -33 | | | | | | | | Cerebellum (Crus I) | | | Left | | | | | | | | |  | | | |
| -11 | | | | | | | -80 | | | | | | | | | -43 | | | | | | | | Cerebellum (Crus II) | | | Left | | | | | | | | |  | | | |
| -33 | | | | | | | -66 | | | | | | | | | -59 | | | | | | | | Cerebellum (Lobule VIII) | | | Left | | | | | | | | |  | | | |
| 6 | | | | | | | -69 | | | | | | | | | -45 | | | | | | | | Cerebellum (Lobule VIII) | | | Right | | | | | | | | |  | | | |
|  | | | | | | |  | | | | | | | | |  | | | | | | | |  | | |  | | | | | | | | |  | | | |
| Statistical Parametric Mapping Activation of Informed Decision-Making | | | | | | | | | | | | | | | | | | | | | | | | | | | | | | | | | | | | | | | |
| Coordinates | | | | | | | | | | | | | | | | | | | | | | | | Region | | | Side | | | | | | | | |  | | | |
| x | | | | | | | y | | | | | | | | | z | | | | | | | | MNI | | |  |  |  |  |  |  |  |  |  |  | | | |
| -19 | | | | | | | -61 | | | | | | | | | -42 | | | | | | | | Cerebellum (Lobule VIII) | | | Left | | | | | | | | |  | | | |
| -25 | | | | | | | -55 | | | | | | | | | -57 | | | | | | | | Cerebellum (Lobule VIII) | | | Left | | | | | | | | |  | | | |
| 21 | | | | | | | -40 | | | | | | | | | -51 | | | | | | | | Cerebellum (Lobule VIII) | | | Right | | | | | | | | |  | | | |
| 10 | | | | | | | -45 | | | | | | | | | -33 | | | | | | | | Cerebellum (Lobule IX) | | | Right | | | | | | | | |  | | | |
| 50 | | | | | | | -54 | | | | | | | | | -38 | | | | | | | | Cerebellum (Crus II) | | | Right | | | | | | | | |  | | | |
| 15 | | | | | | | -20 | | | | | | | | | -35 | | | | | | | | Cerebellum (Lobule III) | | | Right | | | | | | | | |  | | | |
|  | | | | | | |  | | | | | | | | |  | | | | | | | |  | | |  | | | | | | | | |  | | | |
|  | | | | | | |  | | | | | | | | |  | | | | | | | |  | | |  | | | | | | | | |  | | | |
| **Knutson et al. 2003** | | | | | | | | | | | | | | | | |  | | | | | | | |  | | | | | | |  | | | | | | | |
|  | | | | | | | |  | | | | | | | | |  | | | | | | | |  | | | | | | |  | | | | | | | |
| Anticipation: Potential gain vs no outcome | | | | | | | | | | | | | | | | | | | | | | | | | | | | | | | |  | | | | | | | |
| Coordinates | | | | | | | | | | | | | | | | | | | | | | | | | Region | | | | | | | Side | | | | | | | |
| x | | | | | | | | y | | | | | | | | | z | | | | | | | | MNI | | | | | | |  | | | | | | | |
| -1 | | | | | | | | -70 | | | | | | | | | -36 | | | | | | | | Vermis VIII | | | | | | | Left | | | | | | | |
|  | | | | | | | |  | | | | | | | | |  | | | | | | | |  | | | | | | |  | | | | | | | |
| Anticipation: Potential loss vs no outcome | | | | | | | | | | | | | | | | | | | | | | | | |  | | | | | | |  | | | | | | | |
| Coordinates | | | | | | | |  | | | | | | | | |  | | | | | | | | Region | | | | | | | Side | | | | | | | |
| x | | | | | | | | y | | | | | | | | | z | | | | | | | | MNI | | | | | | |  | | | | | | | |
| -1 | | | | | | | | -63 | | | | | | | | | -28 | | | | | | | | Vermis VIII | | | | | | | Left | | | | | | | |
|  | | | | | | | | | | | | | | | | | | | | | | | | | | | | | | | | | | | | | | | |
|  | | | | | | |  | | | | | | | | |  | | | | | | | |  | | |  | | | | | | | | |  | | | |
| **Tanaka et al. 2004** | | | | | | | | | | | | | | | |  | | | | | | | |  | | |  | | | | | | | | |  | | | |
|  | | | | | | |  | | | | | | | | |  | | | | | | | |  | | |  | | | | | | | | |  | | | |
| Brain areas that are involved in immediate reward prediction (SHORT vs. NO) | | | | | | | | | | | | | | | | | | | | | | | | | | | | | | | | | | | | | | | |
| - Significant increase in activity in the medial cerebellum | | | | | | | | | | | | | | | | | | | | | | | | | | | | | | | | | | | | | | | |
| Areas involved in future reward prediction (LONG vs. SHORT) | | | | | | | | | | | | | | | | | | | | | | | | | | | | | | | | | | | |  | | | |
| - A robust increase in activity was observed in the left lateral cerebellum | | | | | | | | | | | | | | | | | | | | | | | | | | | | | | | | | | | | | | | |
| **Remijnse et al. 2005** | | | | | | | | | | | | | | | |  | | | | | | | |  | | |  | | | | | | | | |  | | | |
|  | | | | | | |  | | | | | | | | |  | | | | | | | |  | | |  | | | | | | | | |  | | | |
| Brain regions that reflect the dissociation of stimulus – punishment association and affective switching (FRE > PENS + PRE) | | | | | | | | | | | | | | | | | | | | | | | | | | | | | | | | | | | | | | | |
| Coordinates | | | | | | | | | | | | | | | | | | | | | | | | Region | | | Side | | | | | | | | |  | | | |
| x | | | | | | | y | | | | | | | | | z | | | | | | | | MNI | | |  |  |  |  |  |  |  |  |  |  | | | |
| -15 | | | | | | | -48 | | | | | | | | | -24 | | | | | | | | Cerebellum (Lobule IV, V) | | | Left | | | | | | | | |  | | | |
|  | | | | | | |  | | | | | | | | |  | | | | | | | |  | | |  | | | | | | | | |  | | | |
| **Lie et al. 2006** | | | | | | | | | | | | | | | |  | | | | | | | |  | | |  | | | | | | | | |  | | | |
|  | | | | | | |  | | | | | | | | |  | | | | | | | |  | | |  | | | | | | | | |  | | | |
| Contrasting task C with the high-level baseline (C-HLB) (the neural mechanisms underlying the cognitive components associated with the matching task (of non-identical cards)) | | | | | | | | | | | | | | | | | | | | | | | | | | | | | | | | | | | | | | | |
| Coordinates | | | | | | | | | | | | | | | | | | | | | | | | Region | | | Side | | | | | | | | |  | | | |
| x | | | | | | | y | | | | | | | | | z | | | | | | | | MNI | | |  |  |  |  |  |  |  |  |  |  | | | |
| 26 | | | | | | | -74 | | | | | | | | | -48 | | | | | | | | Cerebellum (Lobule VIIB) | | | Right | | | | | | | | |  | | | |
| -28 | | | | | | | -60 | | | | | | | | | -38 | | | | | | | | Cerebellum (Crus I) | | | Left | | | | | | | | |  | | | |
| 8 | | | | | | | -78 | | | | | | | | | -40 | | | | | | | | Cerebellum (Crus II) | | | Right | | | | | | | | |  | | | |
|  | | | | | | |  | | | | | | | | |  | | | | | | | |  | | |  | | | | | | | | |  | | | |
| B-HLB (additionally involved simple working memory operations and related increased attentional demands) | | | | | | | | | | | | | | | | | | | | | | | | | | | | | | | | | | | | | | | |
| Coordinates | | | | | | | | | | | | | | | | | | | | | | | | Region | | | Side | | | | | | | | |  | | | |
| x | | | | | | | y | | | | | | | | | z | | | | | | | | MNI | | |  |  |  |  |  |  |  |  |  |  | | | |
| 32 | | | | | | | -72 | | | | | | | | | -30 | | | | | | | | Cerebellum (Crus I) | | | Right | | | | | | | | |  | | | |
|  | | | | | | |  | | | | | | | | |  | | | | | | | |  | | |  | | | | | | | | |  | | | |
| Conjunction analysis [(A-HLB) * (B-HLB) * C-HLB)] (neural activations common to all three task conditions) | | | | | | | | | | | | | | | | | | | | | | | | | | | | | | | | | | | | | | | |
| Coordinates | | | | | | | | | | | | | | | | | | | | | | | | Region | | | Side | | | | | | | | |  | | | |
| x | | | | | | | y | | | | | | | | | z | | | | | | | | MNI | | |  |  |  |  |  |  |  |  |  |  | | | |
| 26 | | | | | | | -74 | | | | | | | | | -50 | | | | | | | | Cerebellum (Lobule VIIB) | | | Right | | | | | | | | |  | | | |
|  | | | | | | |  | | | | | | | | |  | | | | | | | |  | | |  | | | | | | | | |  | | | |
| A-C (the complete cognitive gradient across the different tasks) | | | | | | | | | | | | | | | | | | | | | | | | | | | | | | | | | | | |  | | | |
| Coordinates | | | | | | | | | | | | | | | | | | | | | | | | Region | | | Side | | | | | | | | |  | | | |
| x | | | | | | | y | | | | | | | | | z | | | | | | | | MNI | | |  |  |  |  |  |  |  |  |  |  | | | |
| -32 | | | | | | | -70 | | | | | | | | | -46 | | | | | | | | Cerebellum (Crus II) | | | Left | | | | | | | | |  | | | |
| 48 | | | | | | | -72 | | | | | | | | | -42 | | | | | | | | Cerebellum (Crus II) | | | Right | | | | | | | | |  | | | |
| 16 | | | | | | | -90 | | | | | | | | | -34 | | | | | | | | Cerebellum (Crus II) | | | Right | | | | | | | | |  | | | |
| 6 | | | | | | | -40 | | | | | | | | | -24 | | | | | | | | Cerebellum (Vermis I, II) | | | Right | | | | | | | | |  | | | |
| -8 | | | | | | | -60 | | | | | | | | | -42 | | | | | | | | Cerebellum (Lobule IX) | | | Left | | | | | | | | |  | | | |
|  | | | | | | |  | | | | | | | | |  | | | | | | | |  | | |  | | | | | | | | |  | | | |
| A-B (areas corresponded largely to the type II activations) | | | | | | | | | | | | | | | | | | | | | | | | | | | | | | | | | | | |  | | | |
| Coordinates | | | | | | | | | | | | | | | | | | | | | | | | Region | | | Side | | | | | | | | |  | | | |
| x | | | | | | | y | | | | | | | | | z | | | | | | | | MNI | | |  |  |  |  |  |  |  |  |  |  | | | |
| -44 | | | | | | | -60 | | | | | | | | | -50* | | | | | | | | Cerebellum (Lobule VIIB) | | | Left | | | | | | | | |  | | | |
| 6 | | | | | | | -46 | | | | | | | | | -20 | | | | | | | | Cerebellum (Lobule III) | | | Right | | | | | | | | |  | | | |
| *Note. Error in the provided z-coordinate of the contrast A-B. The z-coordinate showed 5, but should actually be 50 according to the region described in the text. | | | | | | | | | | | | | | | | | | | | | | | | | | | | | | | | | | | | | | | |
| **Bjork & Hommer 2007** | | | | | | | | | | | | | | | | | | | | | | | | | | | | | | | | | | | | | |  | |
|  | | | | |  | | | | |  | | | | | | | |  | | | | | | | | | | | | | | |  | | | | |  | |
| Anticipatory activations detected by linear contrasts between cues signaling differing reward probabilities | | | | | | | | | | | | | | | | | | | | | | | | | | | | | | | | | | | | | | | |
|  | | | | |  | | | | |  | | | | | | | |  | | | | | | | | | | | | | | |  | | | | |  | |
| p = 1.0 vs. p = 0.5 | | | | | | | | | |  | | | | | | | |  | | | | | | | | | | | | | | |  | | | | |  | |
| Coordinates | | | | | | | | | | | | | | | | | | Region | | | | | | | | | | | | | | | Side | | | | |  | |
| x | | | | | y | | | | | z | | | | | | | | MNI | | | | | | | | | | | | | | |  | | | | |  | |
| -1 | | | | | -61 | | | | | -16 | | | | | | | | Vermis VI | | | | | | | | | | | | | | | Left | | | | |  | |
|  | | | | |  | | | | |  | | | | | | | |  | | | | | | | | | | | | | | |  | | | | |  | |
| p = 0.5 vs. p = 0 | | | | | | | | | |  | | | | | | | |  | | | | | | | | | | | | | | |  | | | | |  | |
| Coordinates | | | | | | | | | | | | | | | | | | Region | | | | | | | | | | | | | | | Side | | | | |  | |
| x | | | | | y | | | | | z | | | | | | | | MNI | | | | | | | | | | | | | | |  | | | | |  | |
| 4 | | | | | -56 | | | | | -14 | | | | | | | | Vermis IV,V | | | | | | | | | | | | | | | Right | | | | |  | |
|  | | | | |  | | | | |  | | | | | | | |  | | | | | | | | | | | | | | |  | | | | |  | |
| Activation by potential reward-specific to requirement for an instrumental response  Higher-order (*p* = 1.0 vs. *p*=0 response trials) vs. (*p* = 1.0 vs. *p* = 0 non-response trials) | | | | | | | | | | | | | | | | | | | | | | | | | | | | | | | | | | | | | |  | |
| Coordinates | | | | | | | | | | | | | | | | | | Region | | | | | | | | | | | | | | | Side | | | | |  | |
| x | | | | | y | | | | | z | | | | | | | | MNI | | | | | | | | | | | | | | |  | | | | |  | |
| 11 | | | | | -61 | | | | | -16 | | | | | | | | Cerebellum (Lobule VI) | | | | | | | | | | | | | | | Right | | | | |  | |
|  | | | | |  | | | | |  | | | | | | | |  | | | | | | | | | | | | | | |  | | | | |  | |
| Activation time-locked to reward notification  Response conditions: *p* = 1.0 wins vs. *p* = 0 non-wins | | | | | | | | | | | | | | | | | | | | | | | | | | | | | | | | |  | | | | |  | |
| Coordinates | | | | | | | | | | | | | | | | | | Region | | | | | | | | | | | | | | | Side | | | | |  | |
| x | | | | | y | | | | | z | | | | | | | | MNI | | | | | | | | | | | | | | |  | | | | |  | |
| 11 | | | | | -46 | | | | | -26 | | | | | | | | Cerebellum (Lobule IV, V) | | | | | | | | | | | | | | | Right | | | | |  | |
| **Marco-Pallarés et al. 2007** | | | | | | | | | | | | | | | | | | | | | | | |  | | |  | | | | | | |  | | | | | |
|  | | | | | | |  | | | | | | | | |  | | | | | | | |  | | |  | | | | | | |  | | | | | |
| Brain areas presenting significant activity in the contrast positive 4negative feedback trials | | | | | | | | | | | | | | | | | | | | | | | | | | | | | | | | | | | | | | | |
| Coordinates | | | | | | | | | | | | | | | | | | | | | | | | Region | | | Side | | | | | | |  | | | | | |
| x | | | | | | | y | | | | | | | | | z | | | | | | | | MNI | | |  |  |  |  |  |  |  |  | | | | | |
| 32 | | | | | | | -71 | | | | | | | | | -40 | | | | | | | | Cerebellum (Crus II) | | | Right | | | | | | |  | | | | | |
| 15 | | | | | | | -80 | | | | | | | | | -23 | | | | | | | | Cerebellum (Crus I) | | | Right | | | | | | |  | | | | | |
|  | | | | | | |  | | | | | | | | |  | | | | | | | |  | | |  | | | | | | |  | | | | | |
| **Tricomi & Fiez 2008** | | | | | | | | | | | | | | | |  | | | | | | | |  | | |  | | | | | | |  | | | | | |
|  | | | | | | |  | | | | | | | | |  | | | | | | | |  | | |  | | | | | | |  | | | | | |
| Regions Displaying an Accuracy x Time interaction (p < 0.0001) | | | | | | | | | | | | | | | | | | | | | | | | | | | | | | | | | |  | | | | | |
| Coordinates | | | | | | | | | | | | | | | | | | | | | | | | Region | | | Side | | | | | | |  | | | | | |
| x | | | | | | | y | | | | | | | | | z | | | | | | | | MNI | | |  | | | | | | |  | | | | | |
| 13 | | | | | | | -76 | | | | | | | | | -34 | | | | | | | | Cerebellum (Crus II) | | | Bilateral with peak activation in the right hemisphere | | | | | | |  | | | | | |
|  | | | | | | |  | | | | | | | | |  | | | | | | | |  | | |  | | | | | | |  | | | | | |
| Regions displaying a Round × Accuracy × Time interaction (p < 0.0001) | | | | | | | | | | | | | | | | | | | | | | | | | | | | | | | | | | | | | | | |
| Coordinates | | | | | | | | | | | | | | | | | | | | | | | | Region | | | Side | | | | | | |  | | | | | |
| x | | | | | | | y | | | | | | | | | z | | | | | | | | MNI | | |  | | | | | | |  | | | | | |
| -15 | | | | | | | -89 | | | | | | | | | -34 | | | | | | | | Cerebellum (Crus II) | | | Left | | | | | | |  | | | | | |
|  | | | | | | |  | | | | | | | | |  | | | | | | | |  | | |  | | | | | | |  | | | | | |
| **Bischoff-Grethe et al. 2009** | | | | | | | | | | | | | | | | | | | | | | | |  | | |  | | | | | | |  | | | | | |
|  | | | | | | |  | | | | | | | | |  | | | | | | | |  | | |  | | | | | | |  | | | | | |
| Activation peaks within the ROIs for the contrast "positive feedback > uninformative feedback" | | | | | | | | | | | | | | | | | | | | | | | | | | | | | | | | | | | | | | | |
| Coordinates | | | | | | | | | | | | | | | | | | | | | | | | Region | | | Side | | | | | | |  | | | | | |
| x | | | | | | | y | | | | | | | | | z | | | | | | | | MNI | | |  | | | | | | |  | | | | | |
| -36 | | | | | | | -50 | | | | | | | | | -24 | | | | | | | | Cerebellum (Lobule VI) | | | Left | | | | | | |  | | | | | |
| 32 | | | | | | | -46 | | | | | | | | | -22 | | | | | | | | Cerebellum (Lobule VI) | | | Right | | | | | | |  | | | | | |
| Activation peaks within the ROIs for the contrast "negative feedback > uninformative feedback" | | | | | | | | | | | | | | | | | | | | | | | | | | | | | | | | | | | | | | | |
| Coordinates | | | | | | | | | | | | | | | | | | | | | | | | Region | | | Side | | | | | | |  | | | | | |
| x | | | | | | | y | | | | | | | | | z | | | | | | | | MNI | | |  | | | | | | |  | | | | | |
| 32 | | | | | | | -74 | | | | | | | | | -24 | | | | | | | | Cerebellum (Lobule VI → Crus I) | | | Right | | | | | | |  | | | | | |
|  | | | | | | |  | | | | | | | | |  | | | | | | | |  | | |  | | | | | | |  | | | | | |
| **Linke et al. 2010** | | | | | | | | | | | | | | | |  | | | | | | | |  | | |  | | | | | | |  | | | | | |
|  | | | | | | |  | | | | | | | | |  | | | | | | | |  | | |  | | | | | | |  | | | | | |
| Regional BOLD response changes to behavioral switching (final reversal error minus control) | | | | | | | | | | | | | | | | | | | | | | | | | | | | | | | | | | | | | | | |
| Coordinates | | | | | | | | | | | | | | | | | | | | | | | | Region | | | Side | | | | | | |  | | | | | |
| x | | | | | | | y | | | | | | | | | z | | | | | | | | MNI | | |  | | | | | | |  | | | | | |
| -36 | | | | | | | -60 | | | | | | | | | -33 | | | | | | | | Cerebellum (Crus I) | | | Left | | | | | | |  | | | | | |
| -27 | | | | | | | -69 | | | | | | | | | -30 | | | | | | | | Cerebellum (Crus I) | | | Left | | | | | | |  | | | | | |
| -15 | | | | | | | -75 | | | | | | | | | -30 | | | | | | | | Cerebellum (Crus I) | | | Left | | | | | | |  | | | | | |
|  | | | | | | |  | | | | | | | | |  | | | | | | | |  | | |  | | | | | | |  | | | | | |
| **Greening et al. 2011** | | | | | | | | | | | | | | | |  | | | | | | | |  | | |  | | | | | | |  | | | | | |
|  | | | | | | |  | | | | | | | | |  | | | | | | | |  | | |  | | | | | | |  | | | | | |
| Main effect of condition OA>IR, RR, and CTL (the impact of condition (RR, IR, OA, and CTL)) | | | | | | | | | | | | | | | | | | | | | | | | | | | | | | | | | | | | | | | |
| Coordinates | | | | | | |  | | | | | | | | |  | | | | | | | | Region | | | Side | | | | | | |  | | | | | |
| x | | | | | | | y | | | | | | | | | z | | | | | | | | MNI | | |  | | | | | | |  | | | | | |
| -20 | | | | | | | -68 | | | | | | | | | -35 | | | | | | | | Cerebellum (Crus I) | | | Left | | | | | | |  | | | | | |
|  | | | | | | |  | | | | | | | | |  | | | | | | | |  | | |  | | | | | | |  | | | | | |
| **Balsters & Ramnani 2011** | | | | | | | | | | | | | | | | | | | | | | | |  | | |  | | | | | | |  | | | | | |
| Neuronal changes in excitability were found in the right Crus I for the condition in which more automatic processes were present. | | | | | | | | | | | | | | | | | | | | | | | | | | | | | | | | | | | | | | | |
| \| **Bellebaum et al. 2012** \| \| \| \|  \|  \| \|  \| \| --- \| --- \| --- \| --- \| --- \| --- \| --- \| --- \| \|  \| \| \| \| \| \| \| \| \| Activations observed for unexpected vs. expected reward in active and observational learners. Activations in areas of interest are highlighted. \| \| \| \| \| \| \| \| \| Active learners: unexpected < expected reward \| \| \| \| \| \|  \| \| \| Coordinates \| \| \| Region \| \| \| Side \| \| \| x \| y \| z \| MNI \| \| \|  \| \| \| -23 \| -29 \| -30 \| Cerebellum (Lobule IV and V) \| \| \| Left \| \| \| Between-group activation differences for the contrast unexpected > expected reward. Activations in areas of interest are highlighted. \| \| \| \| \| \| \| \| \| Active < observational learners \| \| \|  \| \| \|  \| \| \| Coordinates \| \| \| Region \| \| \| Side \| \| \| x \| y \| z \| MNI \| \| \|  \| \| \| -19 \| -41 \| -19 \| Cerebellum (Lobule IV and V) \| \| \| Left \| \| \| Activations observed for unexpected vs. expected non-reward in active and observational learners. Activations in areas of interest are highlighted. \| \| \| \| \| \| \| \| \| Active learners: unexpected < expected non-reward \| \| \| \| \| \|  \| \| \| Coordinates \| \| \| Region \| \| \| Side \| \| \| x \| y \| z \| MNI \| \| \|  \| \| \| 40 \| -40 \| -35 \| Cerebellum (Crus I) \| \| \| Right \| \| \|  \| \|  \|  \| \| \|  \| \| \| Observational learners \| \| \| unexpected < expected non-reward \| \| \|  \| \| \|  \| \| \| Coordinates \|  \|  \| Region \| \| \| Side \| \| \| x \| y \| z \| MNI \| \| \|  \| \| \| -39 \| -63 \| -31 \| Cerebellum (Crus I) \| \| \| Left \| \| \| 31 \| -51 \| -24 \| Cerebellum (Lobule VI) \| \| \| Right \| \| \| 30 \| -61 \| -33 \| Cerebellum (Crus I) \| \| \| Right \| \| \| -23 \| -54 \| -22 \| Cerebellum (Lobule VI) \| \| \| Left \| \| \| 18 \| -59 \| -38 \| Cerebellum (Lobule VIII) \| \| \| Right \| \| \| 17 \| -81 \| -29 \| Cerebellum (Crus I) \| \| \| Right \| \| \| Between-group activation differences for the contrast unexpected < expected non- reward. Activations in areas of interest are highlighted. \| \| \| \| \| \| \| \| \| Active > observational learners \| \| \| \| \| \|  \| \| \| Coordinates \| \| \| Region \| \| \|  \| \| \| x \| y \| z \| MNI \| \| \|  \| \| \| -5 \| -57 \| -10 \| Cerebellum (Lobule IV and V) \| \| \| Left \| \| \|  \|  \|  \|  \| \| \|  \| \| \| active < observational learners \| \| \|  \| \| \|  \| \| \| Coordinates \| \| \| Region \| \| \| Side \| \| \| x \| y \| z \| MNI \| \| \|  \| \| \| 17 \| -80 \| -18 \| Cerebellum (Lobule VI) \| \| \| Right \| \| \| PE-related activations in active and observational learners. Activations in areas of interest are highlighted. \| \| \| \| \| \| \| \| \| between-group conjunction \| \| \| \| \| \|  \| \| \| Coordinates \| \| \| Region \| \| \| Side \| \| \| x \| y \| z \| MNI \| \| \|  \| \| \| -40 \| -75 \| -33 \| Cerebellum (Crus I) \| \| \| Left \| \| \| 30 \| -56 \| -22 \| Cerebellum (Lobule VI) \| \| \| Right \| \| \|  \| \|  \|  \| \| \|  \| \| \| Active learners \| \|  \|  \| \| \|  \| \| \| Coordinates \| \| \| Region \| \| \| Side \| \| \| x \| y \| z \| MNI \| \| \|  \| \| \| 33 \| -44 \| -35 \| Cerebellum (Lobule VI) \| \| \| Right \| \| \| -9 \| -60 \| -33 \| Cerebellum (Lobule VIII) \| \| \| Left \| \| \| 8 \| -66 \| -33 \| Cerebellum (Lobule VIII) \| \| \| Right \| \| \|  \| \| \| \| \| \|  \| \| \| Observational learners \| \| \| \| \| \| \| Coordinates \| \| \| Region \| \| \| Side \| \| \| x \| y \| z \| MNI \| \| \|  \| \| \| 25 \| -74 \| -34 \| Cerebellum (Crus I) \| \| \| Right \| \| \| -40 \| -75 \| -33 \| Cerebellum (Crus I) \| \| \| Left \| \| \| 4 \| -48 \| -19 \| Cerebellum (Vermis IV and V) \| \| \| Right \| \| \| -9 \| -31 \| -12 \| Cerebellum (Lobule IV and V) \| \| \| Left \| \| | | | | | | | | | | | | | | | | | | | | | | | | | | | | | | | | | | | | | | | |
| **Balsters et al. 2013** | | | | | | | | | | | | | | | |  | | | | | | | |  | | |  | | | | | | |  | | | | | |
|  | | | | | | |  | | | | | | | | |  | | | | | | | |  | | |  | | | | | | |  | | | | | |
| Instruction-Related Activity | | | | | | | | | | | | | | | | | | | | | | | |  | | |  | | | | | | |  | | | | | |
| Main effect of rule cues | | | | | | | | | | | | | | | |  | | | | | | | |  | | |  | | | | | | |  | | | | | |
| Coordinates | | | | | | | | | | | | | | | | | | | | | | | | Region | | | Side | | | | | | | Most active condition | | | | | |
| x | | | | | | | y | | | | | | | | | z | | | | | | | | MNI | | |  | | | | | | |  | | | | | |
| 30 | | | | | | | -60 | | | | | | | | | -34 | | | | | | | | Cerebellum (Crus I) | | | Right | | | | | | | Rules | | | | | |
|  | | | | | | |  | | | | | | | | |  | | | | | | | |  | | |  | | | | | | |  | | | | | |
| First-order rule versus control. | | | | | | | | | | | | | | | | | | | | | | | |  | | |  | | | | | | |  | | | | | |
| Coordinates | | | | | | | | | | | | | | | | | | | | | | | | Region | | | Side | | | | | | | Most active condition | | | | | |
| x | | | | | | | y | | | | | | | | | z | | | | | | | | MNI | | |  | | | | | | |  | | | | | |
| 26 | | | | | | | -78 | | | | | | | | | -50 | | | | | | | | Cerebellum (Crus II/Lobule VIIB) | | | Right | | | | | | | 1stR | | | | | |
|  | | | | | | | | | | | | | | | | | | | | | | | | | | | | | | | | | | | | | | | |
| Second-order rule versus control. | | | | | | | | | | | | | | | | | | | | | | | |  | | |  | | | | | | |  | | | | | |
| Coordinates | | | | | | | | | | | | | | | | | | | | | | | | Region | | | Side | | | | | | | Most active condition | | | | | |
| x | | | | | | | y | | | | | | | | | z | | | | | | | | MNI | | |  | | | | | | |  | | | | | |
| 30 | | | | | | | -60 | | | | | | | | | 32 | | | | | | | | Cerebellum (Crus I) | | | Right | | | | | | | 2ndR | | | | | |
| 10 | | | | | | | -84 | | | | | | | | | -38 | | | | | | | | Cerebellum (Crus II) | | | Right | | | | | | | 2ndR | | | | | |
|  | | | | | | |  | | | | | | | | |  | | | | | | | |  | | |  | | | | | | |  | | | | | |
| SUIT analysis - Main effect of rule cues | | | | | | | | | | | | | | | | | | | | | | | | | | | | | | | | | |  | | | | | |
|  | | | | | | | | | | | | | | | | | | | | | | | | | | | | | | | | | | | | | | | |
|  | | | | | | | coordinates | | | | | | | | | | | | | | | | | | | | cerebellar lobule | | | | | | | most active condition | | | | | |
|  | | | | | | | x | | | | | | | | | y | | | | | | | | z | | |  | | | | | | |  | | | | | |
| Right cerebellum, Crus I | | | | | | | 16 | | | | | | | | | -76 | | | | | | | | -27 | | | Crus I | | | | | | | Rules | | | | | |
| Right cerebellum, Crus I | | | | | | | 34 | | | | | | | | | -74 | | | | | | | | -25 | | | Crus I | | | | | | | Rules | | | | | |
| Right cerebellum, Crus II | | | | | | | 28 | | | | | | | | | -78 | | | | | | | | -53 | | | Crus II; Lobule VIIB | | | | | | | Rules | | | | | |
|  | | | | | | |  | | | | | | | | |  | | | | | | | |  | | |  | | | | | | |  | | | | | |
| 1stR < > 1stC | | | | | | | | | | | | | | | |  | | | | | | | |  | | |  | | | | | | |  | | | | | |
|  | | | | | | | coordinates | | | | | | | | | | | | | | | | | | | | cerebellar lobule | | | | | | | most active condition | | | | | |
|  | | | | | | | x | | | | | | | | | y | | | | | | | | z | | |  | | | | | | |  | | | | | |
| Right cerebellum, Crus I | | | | | | | 44 | | | | | | | | | -66 | | | | | | | | -33 | | | Crus I | | | | | | | 1stR | | | | | |
| Left cerebellum, Crus II | | | | | | | 28* | | | | | | | | | -84 | | | | | | | | -55 | | | Crus II | | | | | | | 1stR | | | | | |
| *Note. Error in the MNI x-coordinate. The provided x-coordinate is positive but the description was focused on the left hemisphere. The “-“ is missing. | | | | | | | | | | | | | | | | | | | | | | | | | | | | | | | | | | | | | | | |
| 2ndR < > 2ndC | | | | | | | | | | | | | | | |  | | | | | | | |  | | |  | | | | | | |  | | | | | |
|  | | | | | | | coordinates | | | | | | | | | | | | | | | | | | | | cerebellar lobule | | | | | | | most active condition | | | | | |
|  | | | | | | | x | | | | | | | | | y | | | | | | | | z | | |  | | | | | | |  | | | | | |
| Right cerebellum, Crus I | | | | | | | 44 | | | | | | | | | -42 | | | | | | | | -33 | | | Crus I | | | | | | | 2ndR | | | | | |
| Right cerebellum, Crus I | | | | | | | 34 | | | | | | | | | -66 | | | | | | | | -29 | | | Crus I | | | | | | | 2ndR | | | | | |
| Right cerebellum, Crus I | | | | | | | 12 | | | | | | | | | -78 | | | | | | | | -23 | | | Crus I | | | | | | | 2ndR | | | | | |
| 1stR < > 2ndR | | | | | | | | | | | | | | | |  | | | | | | | |  | | |  | | | | | | |  | | | | | |
|  | | | | | | | coordinates | | | | | | | | | | | | | | | | | | | | cerebellar lobule | | | | | | | most active condition | | | | | |
|  | | | | | | | x | | | | | | | | | y | | | | | | | | z | | |  | | | | | | |  | | | | | |
| Left cerebellum, Crus I | | | | | | | -46 | | | | | | | | | -68 | | | | | | | | -31 | | | Crus I | | | | | | | 2ndR | | | | | |
| Right cerebellum, Crus I | | | | | | | 32 | | | | | | | | | -64 | | | | | | | | -31 | | | Crus I | | | | | | | 2ndR | | | | | |
| Left cerebellum, Crus I | | | | | | | -32 | | | | | | | | | -82 | | | | | | | | -25 | | | Crus I | | | | | | | 2ndR | | | | | |
| Right cerebellum, Crus I | | | | | | | 8 | | | | | | | | | -80 | | | | | | | | -25 | | | Crus I | | | | | | | 2ndR | | | | | |
|  | | | | | | |  | | | | | | | | |  | | | | | | | |  | | |  | | | | | | |  | | | | | |
| **Lam et al. 2013** | | | | | | | | | | | | | | | |  | | | | | | | |  | | |  | | | | | | |  | | | | | |
|  | | | | | | |  | | | | | | | | |  | | | | | | | |  | | |  | | | | | | |  | | | | | |
| Brain activation during presentation period of card combinations when observing Control Task > WPT Task | | | | | | | | | | | | | | | | | | | | | | | | | | | | | | | | | | | | | | | |
| Coordinates | | | | | | | | | | | | | | | | | | | | | | | | Region | | | Side | | | | | | |  | | | | | |
| x | | | | | | | y | | | | | | | | | z | | | | | | | | MNI | | |  | | | | | | |  | | | | | |
| -46 | | | | | | | -44 | | | | | | | | | -42 | | | | | | | | Cerebellum (Lobule VIIB) | | | Left | | | | | | |  | | | | | |
| 14 | | | | | | | -55 | | | | | | | | | -43 | | | | | | | | Cerebellum (Lobule IX) | | | Right | | | | | | |  | | | | | |
|  | | | | | | |  | | | | | | | | |  | | | | | | | |  | | |  | | | | | | |  | | | | | |
| Activation of cerebral regions observed in comparison of high predictive trials vs. low predictive trials in the feedback period | | | | | | | | | | | | | | | | | | | | | | | | | | | | | | | | | | | | | | | |
| Coordinates | | | | | | | | | | | | | | | | | | | | | | | | Region | | | Side | | | | | | |  | | | | | |
| x | | | | | | | y | | | | | | | | | z | | | | | | | | MNI | | |  | | | | | | |  | | | | | |
| 48 | | | | | | | -44 | | | | | | | | | -47 | | | | | | | | Cerebellum (Crus II) | | | Right | | | | | | |  | | | | | |
|  | | | | | | |  | | | | | | | | |  | | | | | | | |  | | |  | | | | | | |  | | | | | |
| **Späti et al. 2014** | | | | | | | | | | | | | | | |  | | | | | | | |  | | |  | | | | | | |  | | | | | |
| Higher BOLD activity to self-attributed feedback (losses and gains) were found in the cerebellar vermis.  **Kobza & Bellebaum 2015** | | | | | | | | | | | | | | | | | | | | | | | | | | | | | | | | | | | | | | | |
|  | | | | | | |  | | | | | | | | |  | | | | | | | |  | | |  | | | | | | | | |  | | | |
| Cue uncertainty-related activations in observational learners. | | | | | | | | | | | | | | | | | | | | | | | | | | | | | | | | | | | |  | | | |
| Coordinates | | | | | | | | | | | | | | | | | | | | | | | | Region | | | Side | | | | | | | | |  | | | |
| x | | | | | | | y | | | | | | | | | z | | | | | | | | MNI | | |  | | | | | | | | |  | | | |
| 20 | | | | | | | -74 | | | | | | | | | -20 | | | | | | | | Cerebellum (declive) (Lobule VI) | | | Right | | | | | | | | |  | | | |
| 6 | | | | | | | -72 | | | | | | | | | -12 | | | | | | | | Cerebellum (culmen) (Vermis VI) | | | Right | | | | | | | | |  | | | |
| 8 | | | | | | | -74 | | | | | | | | | -20 | | | | | | | | Cerebellum (declive) (Lobule VI) | | | Right | | | | | | | | |  | | | |
| -42 | | | | | | | -60 | | | | | | | | | -26 | | | | | | | | Cerebellum (declive) (Crus I) | | | Left | | | | | | | | |  | | | |
|  | | | | | | |  | | | | | | | | |  | | | | | | | |  | | |  | | | | | | | | |  | | | |
| Action-independent PE-related activations in active learners. | | | | | | | | | | | | | | | | | | | | | | | | | | | | | | | | | | | |  | | | |
| Coordinates | | | | | | | | | | | | | | | | | | | | | | | | Region | | | Side | | | | | | | | |  | | | |
| x | | | | | | | y | | | | | | | | | z | | | | | | | | MNI | | |  | | | | | | | | |  | | | |
| 18 | | | | | | | -70 | | | | | | | | | -38 | | | | | | | | Cerebellum (pyramis) (Lobule VIII) | | | Right | | | | | | | | |  | | | |
| 24 | | | | | | | -80 | | | | | | | | | -26 | | | | | | | | Cerebellum (declive)  (Crus I) | | | Right | | | | | | | | |  | | | |
| 32 | | | | | | | -74 | | | | | | | | | -34 | | | | | | | | Cerebellum (uvula) (Crus I) | | | Right | | | | | | | | |  | | | |
|  | | | | | | |  | | | | | | | | |  | | | | | | | |  | | |  | | | | | | | | |  | | | |
| Action-dependent PE-related activations in active learners | | | | | | | | | | | | | | | | | | | | | | | | | | | | | | | | | | | |  | | | |
| Coordinates | | | | | | | | | | | | | | | | | | | | | | | | Region | | | Side | | | | | | | | |  | | | |
| x | | | | | | | y | | | | | | | | | z | | | | | | | | MNI | | |  | | | | | | | | |  | | | |
| 2 | | | | | | | -66 | | | | | | | | | -24 | | | | | | | | Cerebellum (declive) (Vermis VII) | | | Right | | | | | | | | |  | | | |
| -4 | | | | | | | -70 | | | | | | | | | -16 | | | | | | | | Cerebellum (culmen) (Lobule VI) | | | Left | | | | | | | | |  | | | |
| -6 | | | | | | | -62 | | | | | | | | | -28 | | | | | | | | Cerebellum (declive) (Lobule VIII) | | | Left | | | | | | | | |  | | | |
| -20 | | | | | | | -44 | | | | | | | | | -30 | | | | | | | | Cerebellum (anterior lobule) (Lobule IV, V) | | | Left | | | | | | | | |  | | | |
| -36 | | | | | | | -46 | | | | | | | | | -24 | | | | | | | | Cerebellum (culmen) (Lobule VI) | | | Left | | | | | | | | |  | | | |
|  | | | | | | |  | | | | | | | | |  | | | | | | | |  | | |  | | | | | | | | |  | | | |
| Action-dependent PE-related activations in active and observational learners - active > observational learners | | | | | | | | | | | | | | | | | | | | | | | | | | | | | | | | | | | | | | | |
| Coordinates | | | | | | | | | | | | | | | | | | | | | | | | Region | | | Side | | | | | | | | |  | | | |
| x | | | | | | | y | | | | | | | | | z | | | | | | | | MNI | | |  | | | | | | | | |  | | | |
| 0 | | | | | | | -66 | | | | | | | | | -24 | | | | | | | | Cerebellum (declive) (Vermis VII) | | | Right | | | | | | | | |  | | | |
| -6 | | | | | | | -60 | | | | | | | | | -28 | | | | | | | | Cerebellum (fastigium) (Vermis VIII) | | | Left | | | | | | | | |  | | | |
|  | | | | | | |  | | | | | | | | |  | | | | | | | |  | | |  | | | | | | | | |  | | | |
| **Von der Gablentz et al. 2015** | | | | | | | | | | | | | | | | | | | | | |  | | | | | | |  | | | | | | |  | | | |
|  | | | | | |  | | | | | | |  | | | | | | | | |  | | | | | | |  | | | | | | |  | | | |
| Brain regions showing significant changes in BOLD response for the contrast of switch feedback (switch) versus correct feedback. (switch versus switch n + (2–4)) | | | | | | | | | | | | | | | | | | | | | | | | | | | | | | | | | | | | | | | |
| Coordinates | | | | | | | | | | | | | | | | | | | | | | Region | | | | | | | Side | | | | | | |  | | | |
| x | | | | | | y | | | | | | | z | | | | | | | | | MNI | | | | | | |  | | | | | | |  | | | |
| -28 | | | | | | 60* | | | | | | | -36 | | | | | | | | | Cerebellum (Crus I) | | | | | | | Left | | | | | | |  | | | |
| *Note. Error in the MNI y-coordinate. The provided y-coordinate is positive but the description was focused on the cerebellum. The “-“ is missing. | | | | | | | | | | | | | | | | | | | | | | | | | | | | | | | | | | | |  | | | |
| Brain regions showing significant changes in BOLD response for the contrast of error feedback versus switch | | | | | | | | | | | | | | | | | | | | | | | | | | | | | | | | | | | | | | | |
| Coordinates | | | | | | | | | | | | | | | | | | | | | | Region | | | | | | | Side | | | | | | |  | | | |
| x | | | | | | y | | | | | | | z | | | | | | | | | MNI | | | | | | |  | | | | | | |  | | | |
| 12 | | | | | | -74 | | | | | | | -36 | | | | | | | | | Cerebellum (Crus II) | | | | | | | Right | | | | | | |  | | | |
| -26 | | | | | | -66 | | | | | | | -32 | | | | | | | | | Cerebellum (Crus I) | | | | | | | Left | | | | | | |  | | | |
| 30 | | | | | | -62 | | | | | | | -40 | | | | | | | | | Cerebellum (Crus I) | | | | | | | Right | | | | | | |  | | | |
| 40 | | | | | | -66 | | | | | | | -40 | | | | | | | | | Cerebellum (Crus II) | | | | | | | Right | | | | | | |  | | | |
|  | | | | | | | | | | | | | | | | | | | | | | | | | | | | | | | | | | | |  | | | |
| **Shao et al. 2016** | | | | | | | | | | | | |  | | | | | | | | |  | | | | | | |  | | | | | | |  | | | |
|  | | | | | |  | | | | | | |  | | | | | | | | |  | | | | | | |  | | | | | | |  | | | |
| Neural activations to task-event contrasts of interest (for betting) | | | | | | | | | | | | | | | | | | | | | | | | | | | | | | | | | | | |  | | | |
| Prewin > Preloss | | | | | | | | | | | | |  | | | | | | | | |  | | | | | | |  | | | | | | |  | | | |
| Coordinates | | | | | |  | | | | | | |  | | | | | | | | | Region | | | | | | | Side | | | | | | |  | | | |
| x_max_ | | | | | | y_max_ | | | | | | | z_max_ | | | | | | | | | MNI | | | | | | |  | | | | | | |  | | | |
| -3 | | | | | | -54 | | | | | | | -15 | | | | | | | | | Cerebellum (lobules IV, Vermis IV, V) | | | | | | | bilateral | | | | | | |  | | | |
| Neural activations identified by group-level covariate analyses (for betting) | | | | | | | | | | | | | | | | | | | | | | | | | | | | | | | | | | | | | | | |
| Coordinates | | | | | |  | | | | | | |  | | | | | | | | | Region | | | | | | | Side | | | | | | |  | | | |
| x_max_ | | | | | | y_max_ | | | | | | | z_max_ | | | | | | | | | MNI | | | | | | |  | | | | | | |  | | | |
| -18 | | | | | | -90 | | | | | | | -21 | | | | | | | | | Cerebellum (Crus I) | | | | | | | Left | | | | | | |  | | | |
| Note. x_max,_ y_max_ and z_max_ indicate the MNI coordinates of the peak voxel. | | | | | | | | | | | | | | | | | | | | | | | | | | | | | | | | | | | |  | | | |
| **Peterburs et al. 2018** | | | | | | | | |  | | | | | | | | | |  | | | | | | | | | | | |  | | | | | | | |  |
|  |  | | | | | | | |  | | | | | | | | | |  | | | | | | | | | | | |  | | | | | | | |  |
| Significant clusters for the contrast Neg > Pos. | | | | | | | | | | | | | | | | | | | | | | | | | | | | | | |  | | | | | | | |  |
| Coordinates | | | | | | | | | | | | | | | | | | | Region | | | | | | | | | | | | Side | | | | | | | |  |
| x_max_ | y_max_ | | | | | | | | z_max_ | | | | | | | | | | MNI | | | | | | | | | | | |  | | | | | | | |  |
| -10 | -78 | | | | | | | | -29 | | | | | | | | | | Cerebellum (VIIa /Crus I) | | | | | | | | | | | | Left | | | | | | | |  |
| -8 | -78 | | | | | | | | -27 | | | | | | | | | | Cerebellum (Lobule VI) | | | | | | | | | | | | Left | | | | | | | |  |
|  |  | | | | | | | |  | | | | | | | | | |  | | | | | | | | | | | |  | | | | | | | |  |
| Significant cluster for the contrast Pos1 > FNF. | | | | | | | | | | | | | | | | | | | | | | | | | | | | | | |  | | | | | | | |  |
| Coordinates | | | | | | | | | | | | | | | | | | | Region | | | | | | | | | | | | Side | | | | | | | |  |
| x_max_ | y_max_ | | | | | | | | z_max_ | | | | | | | | | | MNI | | | | | | | | | | | |  | | | | | | | |  |
| -48 | -68 | | | | | | | | -37 | | | | | | | | | | Cerebellum (VIIa / Crus I) | | | | | | | | | | | | Left | | | | | | | |  |
|  |  | | | | | | | |  | | | | | | | | | |  | | | | | | | | | | | |  | | | | | | | |  |
| Significant cluster for the contrast FNF NegStay | | | | | | | | | | | | | | | | | | | | | | | | | | | | | | |  | | | | | | | |  |
| Coordinates | | | | | | | | | | | | | | | | | | | Region | | | | | | | | | | | | Side | | | | | | | |  |
| x_max_ | y_max_ | | | | | | | | z_max_ | | | | | | | | | | MNI | | | | | | | | | | | |  | | | | | | | |  |
| -32 | -74 | | | | | | | | -45 | | | | | | | | | | Cerebellum (VIIa/Crus I) (Voxel 104) | | | | | | | | | | | | Left | | | | | | | |  |
| -12 | -82 | | | | | | | | -33 | | | | | | | | | | Cerebellum (VIIa/Crus I) (Voxel 11) | | | | | | | | | | | | Left | | | | | | | |  |
| -38 | -58 | | | | | | | | -27 | | | | | | | | | | Cerebellum (VIIa/Crus I) (Voxel 112) | | | | | | | | | | | | Left | | | | | | | |  |
| -30 | -70 | | | | | | | | -39 | | | | | | | | | | Cerebellum (VIIa/Crus I) (Voxel 5) | | | | | | | | | | | | Left | | | | | | | |  |
| -10 | -82 | | | | | | | | -25 | | | | | | | | | | Cerebellum (VIIa/Crus I) (Voxel 150) | | | | | | | | | | | | Left | | | | | | | |  |
| -30 | -72 | | | | | | | | -23 | | | | | | | | | | Cerebellum (VIIa/Crus I) (Voxel 50) | | | | | | | | | | | | Left | | | | | | | |  |
| -36 | -48 | | | | | | | | -31 | | | | | | | | | | Cerebellum (Lobule VI) (Voxel 89) | | | | | | | | | | | | Left | | | | | | | |  |
| -10 | -80 | | | | | | | | -25 | | | | | | | | | | Cerebellum (Lobule VI) (Voxel 37) | | | | | | | | | | | | Left | | | | | | | |  |
| -30 | -70 | | | | | | | | -21 | | | | | | | | | | Cerebellum (Lobule VI) (Voxel 2) | | | | | | | | | | | | Left | | | | | | | |  |
| Note. x_max,_ y_max_ and z_max_ indicate the MNI coordinates of the peak voxel. | | | | | | | | | | | | | | | | | | | | | | | | | | | | | | | | | | | | | | |  |
| **Edde et al. 2019** | | | | | | | | |  | | | | | | | | | |  | | | | | | | | | | | |  | | | | | | | |  |
|  |  | | | | | | | |  | | | | | | | | | |  | | | | | | | | | | | |  | | | | | | | |  |
| Young subjects | | | | | | | | |  | | | | | | | | | |  | | | | | | | | | | | |  | | | | | | | |  |
| cerebellar networks (fronto-cerebellar, temporo-cerebellar, cerebello-cerebellar) | | | | | | | | | | | | | | | | | | | | | | | | | | | | | | | | | | | | | | |  |
| Older subjects | | | | | | | | |  | | | | | | | | | |  | | | | | | | | | | | |  | | | | | | | |  |
| sparser modifications of resting-state functional connectivity no cerebellar networks involved | | | | | | | | | | | | | | | | | | | | | | | | | | | | | | | | | | | | | | |  |
|  | | | | | | | | | | | | | | |  | | | | | | | | | | |  | |  |  |  |  |  |  |  |  |  |  |  |  |
|  |  | | | | | | | |  | | | | | | | | | |  | | | | | | | | | | | |  | | | | | | | |  |
| **Jackson et al. 2020** | | | | | | | | | | | | | |  | | | | | | | | |  | | | | | | |  | | | | |  | | | | |
|  | | | |  | | | | | | | | | |  | | | | | | | | |  | | | | | | |  | | | | |  | | | | |
| Areas of peak activation in response to instruction cues on correct trials during all learning blocks compared to control blocks. | | | | | | | | | | | | | | | | | | | | | | | | | | | | | | | | | | | | | | | |
|  | | | |  | | | | | | | | | |  | | | | | | | | |  | | | | | | |  | | | | |  | | | | |
| Young Adult (Region AAL) | | | | | | | | | | | | | | | | | | | | | | |  | | | | | | |  | | | | |  | | | | |
| Coordinates | | | | | | | | | | | | | | | | | | | | | | | Region | | | | | | | Side | | | | |  | | | | |
| x | | | | y | | | | | | | | | | z | | | | | | | | | MNI | | | | | | |  | | | | |  | | | | |
| 30 | | | | -68 | | | | | | | | | | -18 | | | | | | | | | Cerebellum (Lobule VI) | | | | | | | Right | | | | |  | | | | |
| 28 | | | | -30 | | | | | | | | | | -34 | | | | | | | | | Cerebellum (Lobule IV, V) | | | | | | | Right | | | | |  | | | | |
| 50 | | | | -52 | | | | | | | | | | -30 | | | | | | | | | Cerebellum (Crus I) | | | | | | | Right | | | | |  | | | | |
| 34 | | | | -64 | | | | | | | | | | -40 | | | | | | | | | Cerebellum (Crus II) | | | | | | | Right | | | | |  | | | | |
| 32 | | | | -44 | | | | | | | | | | -50 | | | | | | | | | Cerebellum (Lobule VIII) | | | | | | | Right | | | | |  | | | | |
| -48 | | | | -46 | | | | | | | | | | -42 | | | | | | | | | Cerebellum (Crus II) | | | | | | | Left | | | | |  | | | | |
|  | | | |  | | | | | | | | | |  | | | | | | | | |  | | | | | | |  | | | | |  | | | | |
| Old Adult (Region AAL) | | | | | | | | | | | | | | | | | | | | | | | | | | | | | | | | | | |  | | | | |
| Coordinates | | | | | | | | | | | | | | | | | | | | | | | Region | | | | | | | Side | | | | |  | | | | |
| x | | | | y | | | | | | | | | | z | | | | | | | | | MNI | | | | | | |  | | | | |  | | | | |
| 32 | | | | -64 | | | | | | | | | | -18 | | | | | | | | | Cerebellum (Lobule VI) | | | | | | | Right | | | | |  | | | | |
|  | | | |  | | | | | | | | | |  | | | | | | | | |  | | | | | | |  | | | | |  | | | | |
| Areas of peak activation in response to feedback cues during all learning blocks compared to control blocks. | | | | | | | | | | | | | | | | | | | | | | | | | | | | | | | | | | | | | | | |
| Feedback Cue - Learning > Control (YA) | | | | | | | | | | | | | | | | | | | | | | | | | | | | | |  | | | | |  | | | | |
| Coordinates | | | | | | | | | | | | | | | | | | | | | | | Region | | | | | | | Side | | | | |  | | | | |
| x | | | | y | | | | | | | | | | z | | | | | | | | | MNI | | | | | | |  | | | | |  | | | | |
| -26 | | | | -76 | | | | | | | | | | -18 | | | | | | | | | Cerebellum (Lobule VI) | | | | | | | Left | | | | |  | | | | |
| -36 | | | | -80 | | | | | | | | | | -20 | | | | | | | | | Cerebellum (Crus I) | | | | | | | Left | | | | |  | | | | |
| 26 | | | | -84 | | | | | | | | | | -18 | | | | | | | | | Cerebellum (Crus I) | | | | | | | Right | | | | |  | | | | |
| 22 | | | | -76 | | | | | | | | | | -16 | | | | | | | | | Cerebellum (Lobule VI) | | | | | | | Right | | | | |  | | | | |
|  | | | |  | | | | | | | | | |  | | | | | | | | |  | | | | | | |  | | | | |  | | | | |
| Feedback Cue - Learning > Control (OA) | | | | | | | | | | | | | | | | | | | | | | | | | | | | | |  | | | | |  | | | | |
| Coordinates | | | | | | | | | | | | | | | | | | | | | | | Region | | | | | | | Side | | | | |  | | | | |
| x | | | | y | | | | | | | | | | z | | | | | | | | | MNI | | | | | | |  | | | | |  | | | | |
| -40 | | | | -66 | | | | | | | | | | -20 | | | | | | | | | Cerebellum (Lobule VI) | | | | | | | Left | | | | |  | | | | |
| 18* | | | | -30 | | | | | | | | | | -26 | | | | | | | | | Cerebellum (Lobule III) | | | | | | | Left | | | | |  | | | | |
| -40 | | | | -70 | | | | | | | | | | -44 | | | | | | | | | Cerebellum (Crus II) | | | | | | | Left | | | | |  | | | | |
| *Note. Error in the MNI x-coordinate. The provided x-coordinate is positive but the description was focused on the left hemisphere. The “-“ is missing. | | | | | | | | | | | | | | | | | | | | | | | | | | | | | | | | | | |  | | | | |
| Note. All available coordinates were transformed into MNI (where available) space before adding them to the table. *N* = 4 studies did not provide coordinates for the respective cerebellar activation.  In addition, missing regional specification was obtained using the label4MRI package in R and the AAL atlas taxonomy. The description from the text was added if no coordinates were available for the study. | | | | | | | | | | | | | | | | | | | | | | | | | | | | | | | | | | | | | | | |
